# Proteome mapping of epidermal growth factor induced hepatocellular carcinomas identifies novel cell metabolism targets and mitogen activated protein kinase signalling events

**DOI:** 10.1186/s12864-015-1312-z

**Published:** 2015-02-25

**Authors:** Jürgen Borlak, Prashant Singh, Giuseppe Gazzana

**Affiliations:** Centre for Pharmacology and Toxicology, Hannover Medical School, Carl-Neuberg-Str. 1, 30625 Hannover, Germany

**Keywords:** Epidermal growth factor, Transgenic mice, Hepatocellular carcinoma, 2D-PAGE, MALDI-TOF/TOF, Immunhistochemistry, Computational biology

## Abstract

**Background:**

Hepatocellular carcinoma (HCC) is on the rise and the sixth most common cancer worldwide. To combat HCC effectively research is directed towards its early detection and the development of targeted therapies. Given the fact that epidermal growth factor (EGF) is an important mitogen for hepatocytes we searched for disease regulated proteins to improve an understanding of the molecular pathogenesis of EGF induced HCC. Disease regulated proteins were studied by 2DE MALDI-TOF/TOF and a transcriptomic approach, by immunohistochemistry and advanced bioinformatics.

**Results:**

Mapping of EGF induced liver cancer in a transgenic mouse model identified n = 96 (p < 0.05) significantly regulated proteins of which n = 54 were tumour-specific. To unravel molecular circuits linked to aberrant EGFR signalling diverse computational approaches were employed and this defined n = 7 key nodes using n = 82 disease regulated proteins for network construction. STRING analysis revealed protein-protein interactions of > 70% disease regulated proteins with individual proteins being validated by immunohistochemistry. The disease regulated network proteins were mapped to distinct pathways and bioinformatics provided novel insight into molecular circuits associated with significant changes in either glycolysis and gluconeogenesis, argine and proline metabolism, protein processing in endoplasmic reticulum, Hif- and MAPK signalling, lipoprotein metabolism, platelet activation and hemostatic control as a result of aberrant EGF signalling. The biological significance of the findings was corroborated with gene expression data derived from tumour tissues to evntually define a rationale by which tumours embark on intriguing changes in metabolism that is of utility for an understanding of tumour growth. Moreover, among the EGF tumour specific proteins n = 11 were likewise uniquely expressed in human HCC and for n = 49 proteins regulation in human HCC was confirmed using the publically available Human Protein Atlas depository, therefore demonstrating clinical significance.

**Conclusion:**

Novel insight into the molecular pathogenesis of EGF induced liver cancer was obtained and among the 37 newly identified proteins several are likely candidates for the development of molecularly targeted therapies and include the nucleoside diphosphate kinase A, bifunctional ATP-dependent dihydroyacetone kinase and phosphatidylethanolamine-binding protein1, the latter being an inhibitor of the Raf-1 kinase.

**Electronic supplementary material:**

The online version of this article (doi:10.1186/s12864-015-1312-z) contains supplementary material, which is available to authorized users.

## Background

Liver malignancies are common cancers worldwide and are responsible for approximately one million deaths each year with most HCC patients having poor prognosis as a result of rapid disease progression. The relative 5-year survival rate is about 15% and can be attributed to an advanced stage of disease at the time of diagnosis, the occurrence of cirrhosis and of other co-morbidites [1]. Detection of early stages of disease is essential for an improved prognosis and overall survival. However, apart from alpha-fetoprotein (AFP) only a few serological markers are available in clinical practice (such as Glypican-3, miR-21, fucosylated GP73, α-fucosidase) with AFP diagnostics remaining unsatisfactory because of its low sensitivity and the non-specific correlation between the clinical behavior of HCC and AFP blood levels. For this reason, new biomarkers are in strong demand [2,3] and more selective markers, such as soluble interleukin-2 receptor levels, are evaluated [4].

Importantly, research into the molecular pathogenesis of HCC identified several signalling pathways as deregulated. This inspired the development of molecularly targeted therapies such as the multikinase inhibitor sorafenib that inhibits signalling of C-RAF-1, MEK, ERK, VEGFR, PDGFR and other kinases, effectively [5]. Given that epidermal growth factor is an important mitogen for hepatocytes we were particularly interested in an understanding of the consequences of its targeted over-expression in liver. In our initial study we reported the oncogenomics and pathology of EGF induced hepatocarcinogenesis and an identification of molecular circuitries linked to exaggerated EGFR signalling [6]. Furthermore, we investigated the serum proteome of EGF-tumour-bearing mice to obtain information on disease-regulated proteins and to search for novel biomarkers at different stages of disease [7]. Note, a regulatory loop was proposed whereby EGF induces transcriptional activation of HDAC2 by CK2Α/AKT activation in liver cancer cells [8]. Additionally, inhibition of EGFR in different animal models by Erlotinib was shown to attenuate liver fibrosis and the development of hepatocellular carcinoma [9] thus suggesting new therapeutic intervention strategies in the prevention of HCC. Indeed, the EGF receptor tyrosin kinase plays a much wider role in the immortalization of different cell types as originally anticipated [10], and is highly expressed in a number of solid tumours and EGFR over-expression correlates well with tumour progression, resistance to chemotherapy and poor prognosis.

The present study aimed at an identification of disease regulated proteins to facilitate an improved understanding of its complex signalling networks and to search for cross-talk amongst other pathways while an identification of disease regulated proteins would aid the development of molecularly targeted therapies.

For this purpose, a two-dimensional electrophoresis (2-DE) and MALDI-TOF/TOF MS strategy was employed to identify disease regulated proteins in an EGF transgenic mouse model of HCC. This resulted in an identification of 96 statistically significant regulated proteins of which 54 are uniquely expressed in liver cancer. Importantly, 11 out of 54 mouse tumour specific proteins were likewise uniquely expressed in human HCC and 49 disease regulated proteins identified in EGF induced liver cancer were similarly regulated in human HCC, as determined by immunhistochemistry using different antibodies and the information given in the publically available Human Protein Atlas depository. Clinical significance of the identified proteins could be demonstrated and a total of 37 so far unkown proteins could now be related to EGF induced liver cancer, several of which are likely candidates for the development of molecularly targeted therapies. This includes the nucleoside diphosphate kinase A, bifunctional ATP-dependent dihydroxyacetone kinase, phosphatidylethanolamine binding protein1, i.e. an inhibitor of the RAF-1 kinase as well as aldo-keto reductase family 1 proteins, members C14 and C6, interleukin 25 and the v-crk sarcoma virus CT10 oncogene homolog.

Finally, to gain insight into the molecular circuitries of EGFR induced hepatocarcinogenesis diverse computational approaches were employed. This revealed master regulatory proteins and permitted network constructions of 82 disease regulated proteins with protein-protein interactions being confirmed for > 70% of regulated proteins in STRING analysis. Their regulations were also studied by immunohistochemistry in EGF transgenic HCC.

We also compared the serum and liver proteomes of HCC bearing mice and found 10 proteins to be similarly regulated, thus evidencing leakage of tumour proteins that can be detected in serum. Obviously, these are highly interesting biomarker candidates, 6 of which were also regulated in human HCC as determined by immunohistochemistry.

## Methods

### Animal studies

All animal work followed strictly the Public Health Service (PHS) Policy on Humane Care and Use of Laboratory Animals of the National Institutes of Health, USA. Formal approval to carry out animal studies was granted by the animal welfare ethics committee of the State of Lower Saxony, Germany (‘Lower Saxony State Office for Consumer Protection and Food Safety’ (LAVES)). The approval ID is Az: 33.9-42502-04-06/1204.

A total of n = 12 C57/Bl6 non-transgenic and n = 12 EGF transgenic mice (aged 6–8 months), weighing 25–33 g, were housed in Makrolon® Type III cages. Water and food (V1124-000, SSNIFF, The Netherlands) was given *ad libitum*. The temperature and relative humidity was set to 22 ± 2°C and 40–70%, respectively and a 12-h day and night cycle was used.

### Materials and reagents

Tris, urea, thiourea, CHAPS, dithiothreitol, bromophenol blue, glycerin, sodium dodecyl sulfate, glycine, temed, ammoniumperoxodisulfate, ammonium sulfate, ammonium bicarbonate, colloidal Coomassie Blue, and acrylamide were purchased from Roth (Karlsruhe, Germany). Iodacetamide was obtained from SERVA (Heidelberg, Germany) and benzonase was purchased from Novagen (Darmstadt, Germany). Ampholytes (Biolyte 3–10) were purchased from Bio-Rad Laboratories (München, Germany) and DeStreak was obtained from Amersham Bioscience (Freiburg, Germany).

### Mouse liver sample preparation

Mice were anesthetized with ketamine 10% 100 μL/100 g and xylazine 2% 50 μL/100 g, and after surgical removal the liver was perfused and rinsed with ice cold Ringer solution until free of blood.

Approximately 0.1 g of the liver sample was ground in a mortar under liquid nitrogen flow. Then, the samples were processed with 0.5 mL of a buffer containing 40 mM tris base, 7 M urea, 4% CHAPS, 100 mM DTT, and 0.5% (v/v) biolyte 3–10 first (LB2). The suspensions were homogenized by sonication (3 × 20 s) and after addition of 3 μL of benzonase (endonuclease that degrades DNA and RNA) were left at room temperature for 20 min. The samples were then centrifuged at 12,000 g for 20 min. The pellets were washed and sonicated for 5 min with a further 0.5 mL of LB2 and centrifuged at 12,000 g for another 20 min, and the resulting two fractions of supernatant were collected (extract A). Finally, the pellets were redissolved with 0.5 mL of buffer containing 40 mM tris base, 5 M urea, 2 M thiourea, 4% CHAPS, 100 mM DTT, and 0.5% (v/v) biolyte 3–10 (LB3), sonicated, and centrifuged at 12,000 g for 20 min. The pellet was collected, and the supernatant was marked as extract B.

From the same animals, a further 0.1-g portion was ground in a mortar, but was now treated with 0.5 mL of LB3. The suspensions were sonicated, incubated with benzonase, and centrifuged. The pellets were then washed with another 0.5 mL of LB3, sonicated and centrifuged, and the supernatants were collected (extract C).

Proteome mapping was done under a variety of conditions, e.g. extraction with lysis buffers 2 and 3. In addition, proteins were separated at two different pH ranges [5-10]. A total of 4 independent experiments were carried out, and duplicate measurements were run for each experiment. The protein concentration of all extracts was determined using the Bradford assay.

### Liquid-phase IEF pre-fractionation

Liquid-phase IEF pre-fractionation was performed in the Rotofor Cell system (Bio-Rad) following the supplier’s instructions. Ion exchange membranes were equilibrated overnight in the appropriate electrolyte (anion exchange membranes in NaOH 0.1 M and cation exchange membranes in H_3_PO_4_ 0.1 M). After four runs ion exchange membranes were always discarded and new membranes were replaced for the other samples. For each run, the electrode chambers were filled with appropriate fresh electrolytes (30 mL). Initially, the cell was filled with pure water and run for 5 min at 5 watts constant power to remove residual ionic contaminants from the membrane core and ion exchange membranes. Approximately 32 mL of LB2 were used to fill the cell. A total of 60 mg of total proteins in approximately 2 mL of LB2 were added to the cell to reach the maximum loadable volume (40 mL). Focusing started at 12 watts constant power. After approximately 4 hours the voltage increased to 3000 V and the wattage decreased to 3 W. The focused proteins were harvested in 20 ~ 1.5 mL fractions, and pH values were checked. Fractions having pH values between 3 and 7.0 were collected and denoted “A-a” (acid). Fractions having pH values > 7.0 were collected and denoted “A-b” (basic). Again, the protein concentration was determined for both fractions (A-a and A-b) by the Bradford method. Approximately 30 mg of protein were recovered at the end of the liquid-phase IEF pre-fractionation from an initial 60-mg load. The losses are accounted for by the multi-step pre-fractionation procedure, but are not the result of a precipitate that could not be dissolved in our lysis buffer. After each run the membrane core was cleaned with NaOH 0.1 M overnight and sonicated for 5 min in water before the new focusing.

### Two-dimensional gel electrophoresis

#### Isoelectric focusing (IEF) - first dimension

IEF was performed using precast linear IPG strips. The 17-cm IPG strips 7–10 and 5–8 were loaded with 1.5 mg of proteins by active rehydration (12 h, 50 V). Samples destined to be separated by IPG strips 7–10 received an excess of hydroxyethyldisulphide (HED) (DeStreak™) prior to the focusing run. Focusing began at 250 V for 20 min in rapid mode, 10,000 V for 5 h in linear mode and 10,000 V for 50,000 Vh in rapid mode (for the IPG strips 5–8). IEF for the strips 7–10 was carried out at 250 V for 60 min in rapid mode, 10,000 V for 3 h in linear mode and 10,000 V for 50,000 Vh in rapid mode. Each sample was analysed in duplicate. Control and HCC samples were run always at the same time (6 control and 6 HCC samples).

#### 2-DE - second dimension

After IEF, the IPG strips were either stored at −80°C or transferred to 10 mL of equilibration buffer (6 M urea, 30% w/v glycerin, 2% w/v SDS, 50 mM Tris–HCl pH 8.8) with 2% w/v DTT and 0.5% v/v bromophenol blue solution (0.25% w/v bromophenol blue, 1.5 M Tris–HCl pH 8.8, 0.4% w/v SDS) and incubated for 20 min at room temperature. Strips were removed and incubated in equilibration buffer with 4% w/v iodoacetamide and 0.5% v/v bromophenol blue solution for further 20 min at room temperature. Finally, the strips and 10 μL SDS-PAGE molecular weight standard on filter paper were placed on top of the 20 cm x 20.5 cm 12% second-dimension gel (12% v/v acrylamide/bis solution, 375 mM Tris, pH 8.8, 0.1% v/v SDS, 1/2000 TEMED, 0.05% v/v APS). Both were fixed in place with a 0.5% w/v agarose overlay. Gels were run in PROTEAN Plus Dodeca cell (Bio-Rad) at 70 V for approximately 14 h, followed by 200 V until the bromophenol blue dye reached the bottom of the gel. The running buffer (25 mM Tris, 0.2 M glycin, 0.1% SDS) was cooled externally to 16°C.

Gels/proteins were fixed overnight in 30% ethanol, 2% phosphoric acid, and washed 3 x 20 min with 2% phosphoric acid. The gels were equilibrated with 15% ammoniumsulfate, 18% ethanol, 2% phosphoric acid for 15 min and finally stained with colloidal Coomassie Blue for 48 h.

#### Gel scanning and image analysis

After staining, gels were washed 10 min with pure water and scanned on a Molecular FX Scanner (Bio-Rad) at 100 μm resolution. Protein spots were imaged first automatically and then manually and analysed using the PDQuest™ software (Bio-Rad). The normalization was carried out in total density in gel mode according to the manufacturer’s recommendation.

### Matrix-assisted laser desorption ionization mass spectrometry (MALDI-MS)

Gels were excised using the spot cutter of Bio-Rad and placed into 96-well microtiter plates. Excised gel spots were washed manually with 20 μL of water for 10 min and destained twice, first with 15 μL ammonium bicarbonate 50 mM for 5 min and then with 15 μL 50% ammonium bicarbonate 50 mM – 50% acetonitrile for 5 min. Finally, the gel particles were covered by acetonitrile until gel pieces shrunk and left to dry for 10 min. All gels/proteins were digested manually *in situ* with 4 μL of ammonium bicarbonate 50 mM containing 20 ng trypsin (Sequencing Grade Modified Trypsin, Promega, Germany). After 15 min each gel piece was re-swelled with 10 μL of ammonium bicarbonate 50 mM and incubated for 4 h at 37°C. After 4 h the reaction was stopped by adding 10 μL of trifluoroacetic acid 1% containing 1.5% (w/v) n-octyl-beta-D-glucopyranoside (OGP) (AppliChem). For the application of the samples, 4 μL of peptide solution were loaded onto an MTP Anchor Chip Target 600/384 (Bruker Daltonics) previously prepared with a saturated solution of matrix, alpha-cyano-4-hydroxy-cinnamic acid (alpha-HCCA) (Bruker Daltonics, Bremen, Germany).

MALDI-MS was performed on an Ultraflex II MALDI-TOF/TOF (Bruker Daltonics) mass spectrometer equipped with a SmartBeam™ laser and a LIFT-MS/MS facility. The instrument was operated in positive ion reflectron mode and an acceleration voltage of 25 keV for the Peptide Mass Fingerprint (PMF) mode. Typically, 600 spectra, acquired at 100 Hz, were summed and externally calibrated. In the case of MS/MS-CID the LIFT device was used for selection and fragmentation of the ions; the acceleration voltage in the ion source 8 kV, the Timed Ion Selector was set to 0.4% (relative to parent mass), and argon was used as collision gas (~4-6 × 10–6 mbar). Resulting fragments were further accelerated in a second source by 19 kV and analysed by a two-stage gridless reflectron. Typically, 400 shots were accumulated for the parent ion signal and 1000 shots for the fragments. FlexControl™ 3.0, and FlexAnalysis™ 3.0 were used as instrument control and processing software (Bruker Daltonics, Bremen, Germany).

A calibration standard was used for the external calibration of spectra (Peptide Calibration Standard for Mass Spectrometry, which covered the mass range ~1000-4000 Da (Bruker Daltonics). Typically, 1 μL of the peptide calibration standard was spotted on 96 calibration positions of the Anchor Chip Target (Bruker Daltonics) containing the following peptides: angiotensin II (1046.5420 Da), angiotensin I (1296.6853 Da), substance P (1347.7361 Da), bombesin (1619.8230 Da), ACTH clip 1–17 (2093.0868 Da), ACTH clip 18–39 (2465.1990 Da), somatostatin 28 (3147.4714 Da) and OGP 1.5% (w/v).

Internal calibration was achieved using trypsin autolysis products (m/z’s 1045.564, 2211.108 and 2225.119) resulting in a mass accuracy of ≤ 50 ppm. Spectra were collected by the FlexControl software without smoothing or baseline subtraction and a peak resolution higher than 6000 or 7000 a.u. in case of DHB and CHCA matrix-sample preparation, respectively. The spectra were sent to the FlexAnalysis software which labeled the peaks for protein identification by ProteinScape 1.3 or BioTools 3.1 (Bruker Daltonics).

Trypsin autolysis products, tryptic peptides of human keratin and matrix ions were automatically discarded by ProteinScape (mass control list). ProteinScape Score Booster feature was used to improve database search results by automatic iterative recalibrations and background eliminations. Protein scores greater than 53 were considered significant (p <0.05, Mascot) and an annotation as mouse protein as the top candidates was requested in the search when no restriction was applied to the species of origin. Identified proteins were checked individually for further considerations.

For PMF peak picking the snap peak detection algorithm, a signal to noise threshold of 6, maximal number of peaks 100, a quality factor threshold 50 and baseline subtraction TopHat was applied. Peptide masses were searched against the Swiss-Prot database (download 2005–197 228 sequences, 71 501 181 residues) employing the MASCOT server (in-house MASCOT-server, Matrix Sciences Ltd., http://www.matrixscience.com/, revision 2.0.0), taking into account carbamidomethyl of cysteines -Carbamidomethyl (C)- as fixed modification and possible oxidation of methionine -Oxidation (M)- as a variable modification but allowing one missed cleavage. Based on initial data, ion precursors were selected by ProteinScape for tandem MS data acquisition (by LIFT-TOF/TOF, Bruker Daltonics, Bremen, Germany). In the MASCOT MS/MS ions search, the restriction Mammalia was applied with peptide tolerance of (70 ppm and MS/MS tolerance of (0.9 Da (fixed and variable modifications as PMF). The acceptance criteria for PMF-based identification were an individual ions score >27, at least five matching peptides and 10% peptide coverage of the theoretical sequences.

### Immunohistochemistry

Livers, dissected from EGF-overexpressing mice aged between 7–9 months, were fixed in 4% buffered paraformaldehyd and embedded in paraffin. 5 μm thick sections were deparaffinized and rehydrated through a descending alcohol series followed by a 4 min washing step in destilled H_2_O. Subsequently, antigen retrieval was performed in citrate buffer (pH 6) by autoclaving the sections 15 min at 121°C. The Envision kit (Dako, Hamburg, Germany) was used for immunohistochemistry.

The slides were rinsed with destilled H_2_O and after a 5 min incubation step in tris-buffered saline (washing buffer), endogenous peroxidase activity was blocked with DAKO Peroxidase blocking Reagent for 5 min followed by a second washing step. Thereafter, the sections were blocked for 10 min with protein-block serum free (Dako) and incubated with primary antibodies for 45 min. Details of antibody dilutions with washing buffer are given in Additional file 1: Table S1. In the case of goat primary antibodies a rabbit-anti-goat bridging antibody (Dako) was employed. Specifically, the bound primary antibodies or bridging antibodies were detected by use of labelled polymer HRP Anti-Rabbit secondary antibody (Envision Kit; Dako) and the immunoreactivity was visualized by DAKO Liquid DAB Substrate Chromogen System in a 5 min incubation.

Finally, the sections were counterstained with Harris Haematoxylin for 2 min, dehydrated in an ascending alcohol series, coverslipped and examined under a light microscope (Leica, Jülich, Germany).

### Bioinformatic analysis

A total of n = 122 disease regulated proteins were filtered for statistical significance at p < 0.05 (Table 1). This yielded n = 96 statistically significantly regulated proteins two of which had identical accession number, i.e. AAH81431 = ATP synthase H+ transporting mitochondrial F0 complex, subunit d and BAC36241 = APOA1 but differed in their spot IDs as a result of posttranslational modifications. The statistically significantly regulated proteins were grouped into four different categories to yield 54 tumour specific (To), 9 up-regulated (UR), 19 down-regulated (DR) and 14 proteins only expressed in healthy non-transgenic control livers (Co).

**Table 1:** **Disease regulated proteins in HCC of EGF transgenic mice**

**No.**	**Protein**	**GI number (Protein)**	**Accession number (Protein)**	**Gene Symbol**	**Mr**	**pI**	**Mascot score**	**Gels**	**C**	**T**	**LB2**	**LB3**	**p-value**	**Ratio T/C**	**Cellular location**	**References**
1	170 kDa glucose regulated protein GRP170 precursor	7643979	AAF65544	Hyou1	111	5	214	10	0	10	8	2		T	ER, ES	[11,12]
2*	2-hydroxyphytanoyl-CoA lyase	18204150	AAH21360	Hacl1	64	5.9	261	32	19	13	22	10	0.027	0.38	P	
3	3-phosphoglycerate dehydrogenase	52353955	NP_058662	Phgdh	57	6	204	14	0	14	11	3		T		[13]
4*	4931406C07Rik (Ester hydrolase C11orf54 homolog) (spot 4342)	71059921	CAJ18504	4931406C07Rik	35	5.8	184	35	17	18	24	11	0.033	1.5	N	
5*	4931406C07Rik (Ester hydrolase C11orf54 homolog) (spot 4349)	71059921	CAJ18504	4931406C07Rik	35	5.8	195	33	15	18	22	11	0.244	1.9	N	
6	Acylpeptide hydrolase; N-acylaminoacyl peptide hydrolase	22122789	NP_666338	Apeh	80	5.2	182	5	0	5	0	5		T		[14]
7*	Aldo-keto reductase family 1, member C12	15215042	AAH12643	Akr1c12	37	6.1	135	24	14	10	15	9	0.301	1.9		
8	Akr1c18 protein (aldo-keto reductase family 1, member C18)	19527284	NP_598827	Akr1c18	37	5.9	189	11	0	11	5	6		T	C	[15]
9*	Alanyl-tRNA synthetase	34610207	NP_666329	Aars	107	5.4	178	4	0	4	0	4		T	C	
10	Albumin 1 (spot 3707)	33859506	NP_033784	Alb	69	5.7	444	43	21	22	31	12	0.068	3.88	C, ES	[15,16]
11	Albumin 1 (spot 3712)	33859506	NP_033784	Alb	69	5.7	416	40	20	20	30	10	0.25	2.04	C, ES	[15,16]
12	Albumin 1 (spot 4506)	33859506	NP_033784	Alb	69	5.7	423	40	20	20	31	9		T	C, ES	[15,16]
13	Albumin 1 (spot 4702)	33859506	NP_033784	Alb	69	5.7	355	42	20	22	31	11	0.069	1.85	C, ES	[15,16]
14	Albumin 1 (spot 5509)	33859506	NP_033784	Alb	69	5.7	473	38	18	20	30	8	0.392	3.59	C, ES	[15,16]
15*	Aldo-keto reductase family 1, member C14	19527294	NP_598833	Akr1c14	37	5.9	189	11	0	11	5	6		T		
16*	Aldo-keto reductase family 1, member C6	13487925	NP_085114	Akr1c6	37	8.5	120	10	0	10	7	3		T	C	
17	Aldolase 1, A isoform	53733633	AAH83932	Aldoa	39	9.2	213	12	1	11	11	1		T		[17]
18*	Aldolase 3	60687506	NP_033787	Aldoc	39	6.5	153	5	0	5	4	1		T	M	
19	Alpha enolase (spot 4501)	58476212	AAH89539	Gm5506 or Enol1	47	6.4	341	45	22	23	33	12	0.24	0.22	C	[3,13,18,19]
20	Alpha enolase (spot 4516)	58476212	AAH89539	Gm5506 or Enol1	47	6.4	338	40	21	19	30	10	0.161	0.47	C	[3,13,18,19]
21	Alpha enolase (spot 4524)	58476212	AAH89539	Gm5506 or Enol1	47	6.4	338	42	22	20	31	11	0.021	0.53	C	[3,13,18,19]
22	Alpha enolase (spot 5510)	58476212	AAH89539	Gm5506 or Enol1	47	6.4	330	45	22	23	34	11	0.234	0.76	C	[3,13,18,19]
23*	Alpha glucosidase 2	26326711	BAC27099	Ganab	107	5.6	269	5	0	5	0	5		T	ER, G	
24	Annexin A6	31981302	NP_038500	Anxa6	76	5.3	267	5	0	5	0	5		T	C	[14,16]
25	Apolipoprotein A-IV	14789706	AAH10769	Apoa4	45	5.3	224	21	6	15	18	3	0.011	2.77	ES	[20]
26	Apolipoprotein E	71060041	CAJ18564	Apoe	36	5.5	220	31	14	17	21	10	0.01	3.97	ES	[14,21]
27	Apolipoprotein A-I (spot 2215)	26345182	BAC36241	Apoa1	31	5.4	173	38	21	17	28	10	0.001	5.4	S	[13,20,22]
28	Apolipoprotein A-I (spot 3204)	26345182	BAC36241	Apoa1	31	5.4	170	30	18	12				T	S	[20,22]
29	Arginase 1, liver	7106255	NP_031508	Arg1	35	6.6	306	38	23	15	28	10	0.013	0.32	C	[3,13,23,24]
30*	Arginase type II	6753110	NP_033835	Arg2	39	6.1	161	6	0	6	6	0		T	M	
31	Argininosuccinate synthetase 1	6996911	NP_031520	Ass1	47	8.5	175	15	15	0	10	5		C	M	[25-31]
32	ATP synthase, H+ transporting, mitochondrial F0 complex, subunit d (spot 2120)	51980458	AAH81431	Atp5h	19	5.3	148	37	20	17	25	12	0.002	0.33	M	[32]
33	ATP synthase, H+ transporting, mitochondrial F0 complex, subunit d (spot 3203)	51980458	AAH81431	Atp5h	19	5.3	145	35	19	16	22	13		C	M	[32]
34*	Beta 5-tubulin	18088719	AAH20946	Tubb	50	4.7	267	11	1	10	7	4		T	Ck	
35*	Branched chain ketoacid dehydrogenase E1, alpha polypeptide	31982494	NP_031559	Bckdha	50	8.4	237	26	18	8	20	6	0.018	0.5	MM	
36*	Butyryl Coenzyme A synthetase 1	16905127	NP_473435	Acsm1	65	6.6	119	4	4	0	0	4		C	MM	
37	Cai protein (Pdia4)	45219865	AAH66857	Pdia4	65	5.9	267	5	0	5	1	4		T	ER	
38	Capping protein alpha 1 subunit (Capza1)	595917	AAC00566	Capza1	33	5.2	114	11	0	11	7	4		T	Ck	[13]
39	Carbamoyl-phosphate synthetase 1, mitochondrial	8393186	NP_058768	Cps1	165	6.3	350	31	22	9	22	9	0.023	0.2	M, C	[14,15,31,33,34]
40	Carboxylesterase MH1	14331135	BAB60698	Ces1d	62	6.2	168	4	0	4	0	4		T	ER	[17,35]
41*	cDNA sequence BC021917 (dihydroxyacetone kinase 2 homolog)	21703976	NP_663471	Dak	60	6.3	170	4	0	4	1	3		T		
42	Creatine kinase	10946574	NP_067248	Ckb	43	5.3	181	4	0	4	0	4		T	M, C	[13]
43	Cryz protein	13277837	AAH03800	Cryz	35	8.2	84	5	5	0	5	0		C	C	[36]
44	Cu/Zn superoxide dismutase 1513495A	226471	1513495A	Sod1	16	5.9	186	39	21	18	27	12	0.007	2.92		[13,22]
45	DEMSMC malate dehydrogenase, cytosolic	319837	P14152	Mdh1	37	5.9	138	31	17	14	19	12	4E-05	0.27	C	[37]
46*	Dhdh (dihydrodiol dehydrogenase (dimeric)) protein	21618806	AAH31710	Dhdh	37	5.7	209	26	17	9	16	10	1E-04	0.17		
47*	Diacetyl/L-xylulose reductase	50400594	Q91X52	Dcxr	26	7.8	228	5	5	0	0	5		C	MEM	
48*	Dmgdh protein (Dimethylglycine dehydrogenase, mitochondrial)	12836171	BAB23536	Dmgdh	97	7.6	184	8	8	0	5	3		C	M	
49*	Enoyl coenzyme A hydratase 1, peroxisomal	7949037	NP_058052	Ech1	36	7.4	162	26	11	15	21	5	0.026	0.49	M, P	
50	Eukaryotic translation elongation factor 2	33859482	NP_031933	Eef2	95	6.3	208	6	0	6	3	3		T	C	[38]
51	Eukaryotic translation initiation factor 5A (eIF-5A)	124231	NP_001160068	Eif5a	17	5.1	115	10	5	5	7	3	0.106	3.4	C, N	[14]
52*	Farnesyl diphosphate synthetase	19882207	NP_608219	Fdps	41	5.4	158	5	0	5	0	5		T	C	[39]
53*	Fatty acid binding protein 5, epidermal	6754450	NP_034764	Fabp5	15	6.2	149	15	2	13	13	2	0.09	27.9	C	
54	Fibrinogen, alpha polypeptide	33563252	NP_034326	Fga	61	7	144	15	0	15	10	5		T		[31,34]
55	Fibrinogen, B beta polypeptide (spot 5602)	33859809	NP_862897	Fgb	55	6.5	238	35	15	20	23	12	0.001	2.41	ES	[34]
56	Fibrinogen, B beta polypeptide (spot 5612)	33859809	NP_862897	Fgb	55	6.5	244	33	15	18	22	11	0.187	3.54	ES	[34]
57	Fibrinogen, gamma polypeptide	18044708	AAH19828	Fgg	49	5.5	196	12	1	11	5	7		T	ES	[31,40]
58	FK506 binding protein 4	6753882	NP_034349	Fkbp4	52	5.5	154	4	0	4	1	3		T	C, N	[13,14]
59	GDP dissociation inhibitor 2 (GDI 2)	38197560	AAH61767	Gdi2	51	5.8	220	5	0	5	0	5		T	G, C, MEM	[14]
60	Glutathione peroxidase 1	6680075	NP_032186	Gpx1	22	6.2	215	25	18	7	18	7	2E-05	0.28	C, M	[41-43]
61	Glutathione S-transferase, mu 2	6680121	NP_032209	Gstm2	26	7.6	222	11	1	10	7	4		T	C	[14,15]
62	Glycine N-methyltransferase (spot 4256)	34013296	AAL06142	Gnmt	33	6.9	214	23	23	0	17	6		C	C	[15,40,44,45]
63	Glycine N-methyltransferase (spot 5269)	34013296	AAL06142	Gnmt	33	6.9	226	22	22	0	15	7		C	C	[15,40,44,45]
64	Glycine N-methyltransferase (spot 9105)	34013296	AAL06142	Gnmt	33	6.9	218	20	20	0	15	5		C	C	[15,40,44,45]
65	Glycyl-tRNA synthetase	21264024	Q9CZD3	Gars	82	6.2	180	5	0	5	0	5		T	C	[46]
66	Haao protein (3-hydroxyanthranilate 3,4-dioxygenase)	15277547	AAH12872	Haao	33	6	420	35	22	13	27	8	0.006	0.24	C	[14]
67	Hal (histidine ammonia lyase) protein	35505393	AAH57637	Hal	72	5.9	301	6	6	0	0	6		C	C	[47]
68	Hemopexin	23956086	NP_059067	Hpx	51	9	231	16	2	14	11	5	0.323	7.27	ES	[48]
69	Heterogeneous nuclear ribonucleoprotein L (hnRNPL)	33667042	NP_796275	Hnrnpl	60	6.6	156	8	0	8	8	0		T	N	[13]
70	HSP60 (spot 2604)	1334284	CAA37654	Hspd1	58	5.3	298	15	7	8	6	9	0.008	0.4	MM	[13,14,35]
71	HSP60 (spot 2610)	1334284	CAA37654	Hspd1	58	5.3	298	14	7	7	6	8	0.012	0.5	MM	[13,14,35]
72*	Hypothetical protein LOC68347	58037115	NP_080962	0610011F06Rik	23	5.8	125	29	15	14	21	8	0.014	0.39		
73*	Inosine triphosphatase	31982664	NP_080198	Itpa	22	5.4	139	5	0	5	5	0		T	C	
74	Interleukin 1 receptor antagonist protein	238585	AAB20265	Il1rn	18	5.5	121	7	0	7	6	1		T	S, C	[35-37,41-43,45-50]
75*	Interleukin 25 or UPF0556 protein C19orf10 homolog precursor	18250288	NP_543027	D17Wsu104e	18	5.9	89	13	6	7	9	4	0.23	2.1	ES	
76*	Kininogen 1	12963497	NP_075614	Kng1	48	5.7	217	21	8	13	19	2	0.091	2.8	ES	
77	Lamin-A/C	15929761	AAH15302	Lmna	74	6.6	199	12	0	12	10	2		T	N	[13,15]
78*	LIM and SH3 protein 1	6754508	NP_034818	Lasp1	30	6.7	156	7	0	7	6	1		T	Ck	
79	Liver fructose-1,6-bisphosphatase	6688689	CAB65244	Fbp1	37	6.2	281	29	18	11	17	12	0.013	0.35		[14,34,40]
80*	Lysophospholipase 1	6678760	NP_032892	Lypla1	25	5.9	134	25	13	12	20	5	0.25	0.25	M	
81	Major urinary protein	1839508	AAB47130	Mup14	19	4.7	138	6	6	0	6	0		C	S	[51,52]
82*	Major vault protein	12003287	AAG43520	Mvp	96	5.4	238	4	0	4	0	4		T	C	
83	MAWD binding protein homolog 1 or Phenazine biosynthesis-like domain-containing protein 1	31560132	NP_080977	Pbld1	32	5.1	318	25	19	6	19	6	0.065	0.38		[3,14,40]
84*	Mitochondrial acyl-CoA thioesterase 1 OR Acyl-CoA thioesterase 2	40538846	NP_598949	Acot2	50	7	123	6	6	0	1	5		C	M	
85	NADH dehydrogenase (ubiquinone) 1 alpha subcomplex, 8	21312012	NP_080979	Ndufa8	20	8.8	145	4	4	0	4	0		C	M	[34,53]
86	NADH dehydrogenase (ubiquinone) Fe-S protein 1 (Ndufs1)	26331822	BAC29641	Ndufs1	80	5.5	240	5	5	0	0	5		C	M	[14]
87	NADH dehydrogenase (ubiquinone) flavoprotein 1	19526814	NP_598427	Ndufv1	51	8.5	151	4	0	4	3	1		T	M, ES	[53]
88	Nit protein 2 (spot 5315)	12963555	NP_075664	Nit2	31	6.2	347	34	18	16	24	10		C		[3,13]
89	Nit protein 2 (spot 6201)	12963555	NP_075664	Nit2	31	6.2	345	30	16	14	22	8	0.295	0.8		[3,13]
90*	Nucb1 (nucleobindin 1) protein	49117484	AAH72554	Nucb1	53	5	184	8	0	8	8	0		T	G, C, MEM	
91	Peroxiredoxin 6 (spot 4207)	6671549	NP_031479	Prdx6	25	5.7	282	40	21	19	30	10	0.132	2.3	C, L	[3]
92	Peroxiredoxin 6 (spot 5216)	6671549	NP_031479	Prdx6	25	5.7	280	38	20	18	28	10	0.018	0.3	C, L	[3]
93	Phosphatidylethanolamine binding protein	53236978	AAH83063	Pebp1	21	5.2	135	12	2	10	11	1	0.008	2.2	C	[22,40,53]
94	Plasminogen	200403	AAA50168	Plg	91	6	243	9	0	9	6	3		T	S	[33,54-56]
95*	Poly(rC) binding protein 2; heterogeneous nuclear ribonucleoprotein X or Poly(rC) binding protein 1	6754994	NP_035995	Pcbp1	38	6.4	189	8	0	8	8	0		T	N	
96	Agmatine ureohydrolase (agmatinase)	20848362	XP_131722	Agmat	38	8	108	24	16	8	19	5	0.003	0.1	ES	
97	Prohibitin	54035592	AAH83354	Phb	30	5.4	335	39	21	18	28	11	0.018	0.6	M	[13]
98*	Psmd11 (proteasome (prosome, macropain) 26S subunit, non-ATPase, 11) protein	33585718	AAH55457	Psmd11	47	6.1	258	4	0	4	0	4		T	C	
99	Pyridoxine 5′-phosphate oxidase	19527238	NP_598782	Pnpo	30	8.4	120	24	14	10	16	8	0.026	0.3		[15,40]
100	Pyruvate kinase 3 or pyruvate kinase, muscle isoform M2	31981562	NP_035229	Pkm	58	7.9	274	7	0	7	4	3		T	M	[57]
101	Pzp protein (a2-macroglobulin) or Pregnancy zone protein	34785996	AAH57983	Pzp	166	6.2	151	14	2	12	9	5		T	ES	[58]
102	Retinol binding protein 4, plasma	33859612	NP_035385	Rbp4	23	5.6	144	19	2	17	14	5	0.129	12.7	ES	[3]
103*	RIKEN cDNA 1810013B01 (abhydrolase domain containing 14b)	27753960	NP_083907	Abhd14b	23	5.6	172	31	16	15	23	8	0.042	0.6	N, C	
104*	RIKEN cDNA 2410004H02 or Aldehyde dehydrogenase family 16 member A1	26080429	NP_666066	Aldh16a1	85	5.8	114	4	0	4	0	4		T		
105*	Ribosomal protein S12	34849622	AAH58460	Rps12	15	7.3	90	8	1	7	6	2	0.008	2	C	
106*	Sars1 protein or Seryl-aminoacyl-tRNA synthetase	14250361	AAH08612	Sars	58	5.9	199	4	0	4	0	4		T	C	
107	Selenium binding protein 1	22164798	NP_033176	Selenbp1	53	5.9	336	31	19	12	21	10	0.061	0.5	C, MEM, N	[14]
108	Serine (or cysteine) proteinase inhibitor, clade B, member 6a	6678097	NP_033280	Serpinb6a	43	5.4	137	5	0	5	0	5		T	C	[13]
109*	Serpinb1a protein	12834891	BAB23079	Serpinb1a	43	5.7	255	8	8	0	4	4		C	C	
110	Serum amyloid P-component	38174334	AAH61125	Apcs	26	6	141	4	0	4	0	4		T	ES	[14-16]
111*	Sorcin	13385076	NP_079894	Sri	20	4.9	129	7	0	7	6	1		T	C, MEM	
112*	T43799 proteasome protein p45/SUG [imported]	11265288	P62198	Psmc5	46	7.6	222	6	0	6	6	0		T	C, N	
113	T-complex protein 1, theta subunit (TCP-1-theta) (CCT-theta)	12846632	BAB27244	Cct8	50	5.5	189	5	0	5	0	5		T	C	[15]
114	Transglutaminase 2, C polypeptide	6678329	NP_033399	Tgm2	77	5	240	6	0	6	3	3		T	C, MEM	[14]
115	Transthyretin	7305599	NP_038725	Ttr	16	5.5	127	18	6	12	12	6	0.169	3	ES	[16,20]
116	Tumor metastatic process-associated protein NM23	51980604	AAH82178	Nme1	17	6.4	134	9	0	9	7	2		T		[13,14]
117*	Uap1l1 (UDP-N-acteylglucosamine pyrophosphorylase 1-like 1) protein	28175154	AAH43307	Uap1l1	57	5.2	168	4	0	4	0	4		T		
118	UDP-glucose dehydrogenase	6678499	NP_033492	Ugdh	55	7.4	307	14	0	14	13	1		T		[46]
119	Galectin-3 or Lectin, galactose binding, soluble 3	52987	CAA34736	Lgals3	28	8.8	136	6	0	6	5	1		T	N, C	[13]
120*	v-crk sarcoma virus CT10 oncogene homolog	56205173	CAI24083	Crk	34	5.3	187	8	0	8	8	0		T	C, MEM	
121	Vimentin	31982755	NP_035831	Vim	54	5	260	26	11	15	21	5	0.022	4	Ck	[13,35]
122	Vitamin D-binding protein	193446	AAA37669	Gc	53	5.2	229	32	15	17	22	10	0.175	1.6	ES	[59,60]

*Abbreviations: C, cytosol; Ck, cytoskeleton; M, mitochondria; N, nucleus; P, peroxisome; ES, extracellular space; ER, endoplasmic reticulum; G, golgi; L, lysosome; MEM, membrane; MM, mitochondrial matrix; S, secreted.*

The proteins are sorted in alphabetical order, and the NCBI annotation is given in the accession number column. Molecular weight, pI, and MASCOT scores are also given. The column “Gels”, “C” (C = control) and “T” (T = tumour) indicate the frequency of positive identification of proteins in a total of 48 independent gels, whereas “LB2” and “LB3” (LB = lysis buffer) refers to the different lysis buffers employed. Furthermore, references are given for those proteins which have already been described as HCC-associated whereas those marked with a star (*) are so far unknown as EGFR disease regulated in hepatocellular carcinoma.

### Categorization of tumour regulated proteins based on ontology terms

82 non-redundant tumour proteins covering To, UR and DR categories were considered and analysed for Ontologies using the GeneXplain software (v.2.4.1), the biological pathways tools Reactome (http://www.REACTOME.org) and KEGG (http://www.genome.jp/kegg) and WikiPathways (http://wikipathways.org). The tumour regulated proteins (To + UR + DR) were subjected to functional classification based on ontology terms and a p-value of <0.01 was considered to be significant. Moreover, disease regulated proteins were analysed with the Cytoscape software version 3.0.2 using the function GO-tree levels and number or % of proteins for a given term (see Additional file 2: Table S2).

### Identification of master regulatory molecules and protein network construction for tumour proteins

Master regulatory proteins were searched based on the designated workflow of the GeneXplain software. It is designed to find master regulatory molecules upstream of an input list of regulated tumour proteins. After annotation of the input datasets the tool for master regulator finding over GeneWays network (http://www.genexplain.com) was applied. Specifically, the GeneWays software is used to automatically extract, analyse, visualise and integrate molecular pathway data from the published peer reviewed literature. It is based on document sorting, term identification, term meaning disambiguation, information extraction, ontology, visualization and system integration [61]. The following filtering threshold was used, i.e. score cutoff (0.2), search collection (GeneWays hub), maximum radius [4,10], FDR cutoff (0.05), Z-score cutoff (1.0), Penalty (0.1) and Decay factor (0.1) (Additional file 3: Table S3).

Protein network for disease regulated proteins were also constructed using the STRING software (http://string-db.org/). The underlying database informs on known and predicted protein-protein interaction and the constructed networks are based on active prediction methods of Neighborhood, Gene Fusion, Co-occurrence, Co-expression, Databases and Textmining. Eventually, confidence scores were calculated for each interaction pair and only those above default cutoff scores (0.4) were selected.

Finally, mapping of pathways information from REACTOME, KEGG and WikiPathways have been implemented over protein networks using information of known pathways and sustained proteins connecting these pathways in a given network.

## Results

**Figure 1:**
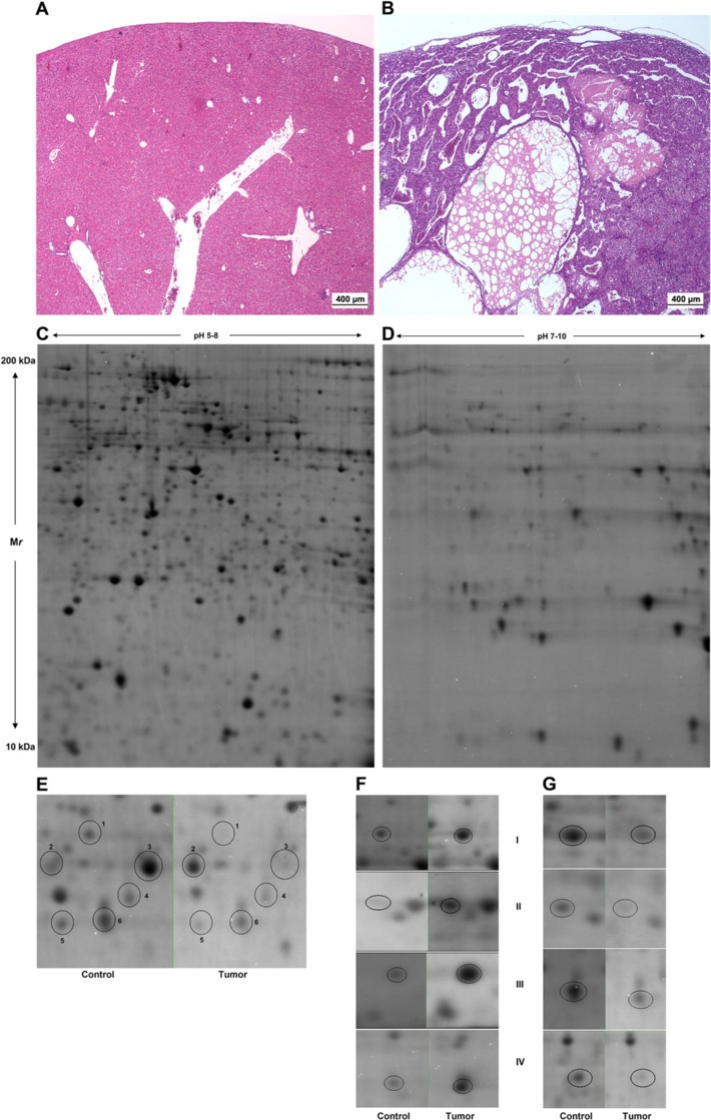
**Liver proteome mapping of healthy non-transgenic control and EGF transgenic mice. (A)** Histopathology of a well-organized normal liver tissue of a non-transgenic control (image magnification 25-fold). **(B)** Histopathology of completely disorganized tissue of advanced HCC showing multilayered hepatocytic trabeculae besides solid areas, peliosis-like intratumourous vasectasias and focal necroses (light red) (image magnification 25-fold). **(C)** Zoom-in 2D gel image of healthy non-transgenic control liver extracts in the pH range of 5–8. **(D)** Zoom-in 2D gel image of healthy non-transgenic control liver extracts in the pH range of 7–10.Panel E to G depict examples of zoom-in 2D gels of regulated proteins. **(E)** Spot 1: glycine N-methyltransferase, identified in control samples only; Spot 2: peroxiredoxin 6, up-regulated in tumour samples; Spot 3: peroxiredoxin 6, down-regulated in tumour samples; Spot 4: lysophospholipase 1, down-regulated in tumour samples; Spot 5: hypothetical protein LOC68347, down-regulated in tumour samples; Spot 6: glutathione peroxidase 1, down-regulated in tumour samples. **(F)** Examples of up-regulated mouse liver proteins: fibrinogen β (I), vimentin (II), Cu/Zn superoxide dismutase (III), and apolipoprotein E (IV). **(G)** Examples of down-regulated mouse liver proteins: arginase 1 (I), Dhdh protein (II), glutathione peroxidase 1 (III) and predicted: agmatine ureohydrolase (IV).

The histopathology and oncogenomics of EGF induced liver cancers was previously reported [6] and an important finding of the study was the 100% incidence of malignant tumour formation in less than one year after birth. Notably, a sequence of events was observed that initially consisted of diffuse large cell dysplasia followed by multiple dysplastic foci and nodules and growth of HCC. Figure 1 A and B depict the histopathology of healthy non-transgenic control liver and EGF induced tumours, respectively.

### Image analysis of differentially expressed proteins

After protein extraction 2DE was performed. Subsequently, the gels were scanned on a Bio-Rad Molecular FX Scanner at a 100 μm resolution. Image analysis was done with the PDQuestTM software and spots were detected automatically. A total of 122 proteins differed in expression or were de novo expressed when 2DE gels of non-transgenic controls and HCC mice were compared (see Table 1 for detailed information on the proteins identified and Figure 1E-G depicting examples of zoom-in-gels of some regulated proteins). Among them are 96 statistically significantly regulated proteins (*p* ≤ 0.05) of which 63 were significantly up-regulated (ratio_HCC/control_ ≥ 2) and included fibrinogen and subunits of it, vimentin, Cu/Zn superoxide dismutase, and apolipoprotein E (Figure 1F (I-IV), while 33 proteins were repressed in expression (ratio_HCC/control_ ≤ 0.6) and included arginase 1, Dhdh protein, glutathione peroxidase 1 and agmatine ureohydrolase (Figure 1G (I-IV) and Table 1).

### Identification of proteins by MS analysis

A reference 2-DE map of mouse liver and serum proteins was constructed that consists of more than n = 500 proteins [2,7]. Note, in our previous efforts we identified n = 25 serum proteins as regulated in the EGF transgenic disease model. Among them were alpha-fetoprotein, clusterin, fibrinogen-α and -γ, serum amyloid component P and several apolipoproteins all of which were significantly up-regulated. Based on the combined use of 2DE and MALDI-MS a total of n = 122 differentially expressed proteins were identified (Table 1) and included isoforms as well as post translational modifications of albumin (5 up-regulated spots), alpha enolase (4 down-regulated spots), apoliproptein A-I (2 up-regulated spots), ATP synthase H+ transporting mitochondrial (2 down-regulated spots), fibrinogen beta (2 up-regulated spots), glycine N-methyltransferase (3 spots, in controls only), hsp60 (2 down-regulated spots), nit protein 2 (2 down-regulated spots), peroxiredoxin 6 (1 up-regulated spot and 1 down-regulated spot), and 4931406C07Rik (2 up-regulated spots) (see Table 1). Importantly, a total of n = 37 so far unknown disease regulated proteins were identified that can now be related to EGF induced liver cancer. These are marked with an asterisk in Table 1.

**Figure 2:**
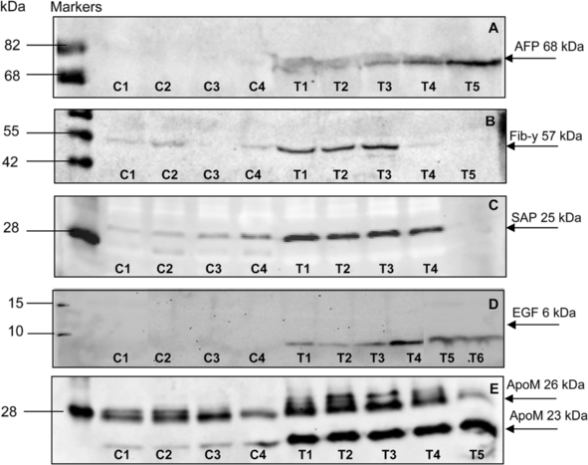
**Western blotting of serum proteins in control and EGF transgenic mice.** For the commonly regulated proteins in serum and tumours their regulation in liver tissue was confirmed by 2DE and MALDI-TOF/MS (see Table 1). Depicted are Western blots for serum proteins. Note, with the exception of EGF the regulated serum proteins were already reported in our earlier publication [7]. C 1–4 = individual control animals, T 1–6 = individual tumour bearing mice. **(A)** alpha-fetoprotein, **(B)** fibrinogen gamma, **(C)** serum amyloid component P, **(D)** epidermal growth factor, **(E)** and apolipoprotein M which was identified in serum samples only.

Furthermore, a comparison of serum and liver proteoms revealed n = 10 proteins to be regulated in common, thus evidencing leakage of tumour proteins into systemic circulation (Table 2). Among them was serum AFP; it’s up-regulation and that of others was confirmed by Western blot analysis (Figure 2A-E). Likewise, apolipoprotein E was up-regulated both in serum and tumour samples, the ratio HCC/control being 2.2 and 3.9, respectively. In a previous study on human HCC increased expression of ApoE was observed in 88% of study cases; however, gene ApoE expression and serum levels were unchanged to suggest its accumulation and impaired secretion [21]. Two isoforms of alpha-2-macroglobulin were up-regulated in serum of HCC-bearing mice (spot 1: ratio_HCC/control_ = 1.8; spot 2: ratio_HCC/control_ = 3.2). Its expression was exclusively associated with tumours. Finally, serum amyloid component P was up to 10-fold up-regulated in serum and its tissue expression was tumour specific (Table 2).

**Table 2:** **Proteins regulated in common in tumour tissue and serum of EGF transgenic mice**

**No.**	**Protein**	**Accession number**	**Ratio**_**HCC/control**_ **(serum)**	**Ratio**_**HCC/control**_ **(liver)**
1	Alpha-fetoprotein	gi|42542817	2	up (by Western blot)
2	Apolipoprotein A1	gi|26345182	tumour	Spot 1: 5.4 Spot 2: tumour
3	Apolipoprotein E	gi|6753102	2.2	3.9
4	Carboxylesterase precursor	gi|2921308	tumour	tumour
5	Fibrinogen, alpha polypeptide	gi|33563252	tumour	tumour
6	Fibrinogen, beta polypeptide	gi|33859809	tumour	Spot 1: 2.4
				Spot 2: 3.5
7	Fibrinogen, gamma polypeptide	gi|19527078	tumour	tumour
8	Major urinary protein 1	gi|8569601	0,1	control
9	Pzp (A2mg protein)	gi|34785996	Spot 1: 1.8	tumour
			Spot 2: 3.2	
10	Serum amyloid P-component	gi|38174334	10	tumour

### Immunohistochemistry of disease-regulated proteins

**Figure 3:**
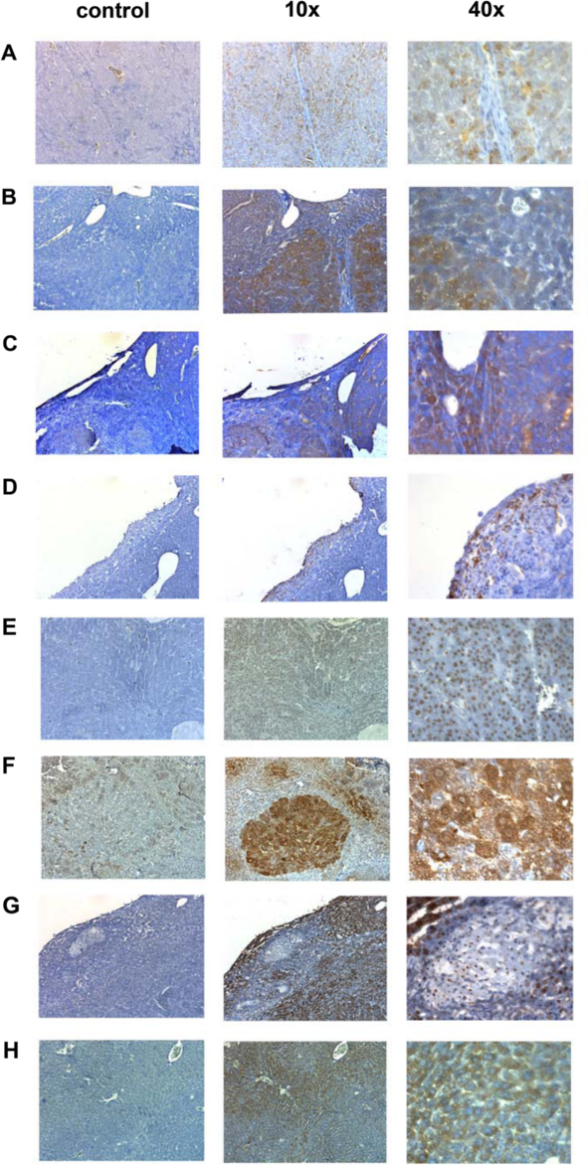
**Immunohistochemistry of proteins regulated in hepatocellular carcinoma of EGF transgenic mice.** Shown are images of **(A)** Arginase II, **(B)** Capza1, **(C)** GDI 2, **(D)** Tubulin β, **(E)** hnRNP L, **(F)** Amphiregulin, **(G)** HNF4α and **(H)** Epiregulin. Specificity was determined by treating the specimen with washing buffer instead of primary antibody (controls); in the case of amphiregulin the specificity was confirmed with a blocking peptide.

To further evidence disease regulated proteins and to provide information on their subcellular localization a total of n = 8 proteins were studied by immunohistochemistry. Five of them were selected for their novelty (see Table 1) while amphiregulin and epiregulin were chosen for their importance in the EGF-signalling pathway. Furthermore, HNF4α was studied for its pivotal role in liver cancer [62]. Depicted in Figure 3 are immunohistochemistry stainings performed with EGF transgenic livers to confirm regulation and predominant cytoplasmic expression of arginase II. Note, ARG2 is only expressed in HCC and recent evidence suggest modulation of arginine levels in the extracellular milieu to be part of an immune escape mechanism whereby lack of local arginine weakens tumour-infiltrating lymphocytes as T cells require adequate argine levels [63]. Likewise, the tumour specific and cytoplasmic expression of the F-actin capping protein α1 subunit (CAPZA1) and the predominant nuclear expression of tubulin β that was particularly visible beneath the liver capsule may possible promote microtubule stability and interactions of microtubules with endogenous proteins. Furthermore, the induced and predominat cytoplasmic expression of the GDP dissociation inhibitor 2 (GDI2) protein is part of the control of vesicular trafficking. This protein is known to regulate GDP-GTP exchange amongst members of the Rab family of proteins. The tumour specific and cytoplasmic expression of amphiregulin supports the notion of a switch in autocrine signalling and it has been reported that amphiregulin is a prognostic marker for poor outcome of a variety of malignancies including colorectal liver metastasis [64]. Finally, the repressed nuclear expression of HNF4A was not unexpected and confirms earlier findings [62].

### Comparison of disease regulated proteins in mouse and human HCC

Based on the information given in Table 1 the Human Protein Atlas depository (www.proteinatlas.org, version 12) was interrogated. As shown in Additional file 4: Table S4 48 out of 96 mouse liver cancer regulated proteins were likewise regulated in human HCC. It should be noted that for some proteins several antibodies were used to study their expression; only representative data were considered. Importantly, out of the 54 proteins uniquely expressed in mouse liver tumours n = 11 were likewise uniquely expressed in human HCC thus evidencing clinical significance of our findings.

### Comparison of gene and protein expression in EGF induced liver cancer

We compared our previously published transcriptomic data of EGF induced liver cancers with the proteomic data obtained in the present study. Such comparisons revealed n = 22 genes to be significantly regulated of which n = 17 are in common regulated whereas for n = 5 genes transcript expression was opposite to that of the coded proteins (see Additional file 5: Table S5).

### Classifications of disease regulated protein by Gene Ontology (GO)

**Figure 4:**
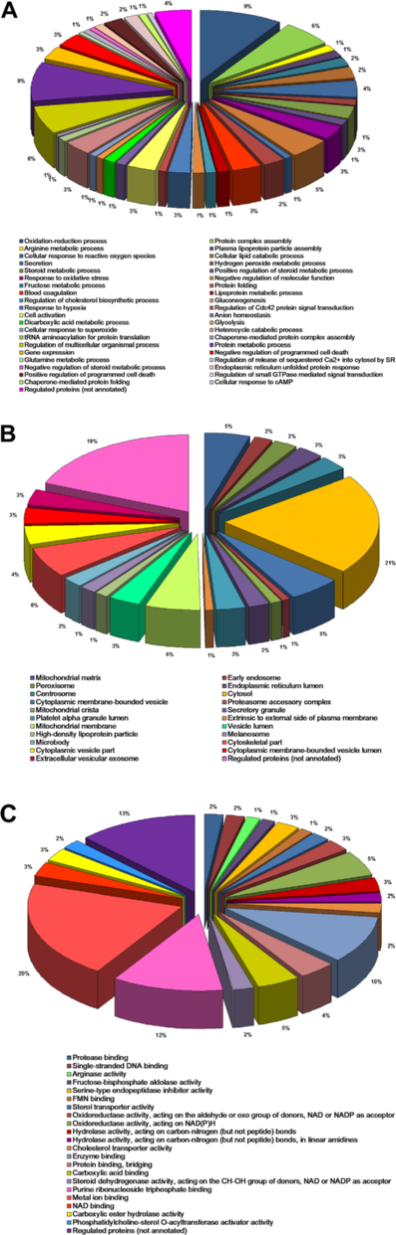
**Significantly regulated proteins categorized by GO terms. (A)** Biological process, **(B)** Cellular components, **(C)** Molecular functions. The pie-charts depict the percentage of proteins involved in the various GO terms.

82 of the 96 significantly regulated proteins were mapped to 40 different biological processes (see Figure 4A) of which prominent examples are regulation of arginine metabolism and amino acid import, regulation of CDC42 protein signal transduction’, cellular response to oxidative stress, hydrogen peroxide and superoxide, glycolysis and gluconeogenesis, regulation of cholesterol transport, protein-lipid complex and plasma lipoprotein particle remodeling, positive regulation of steroid metabolic process, negative regulation of calcium ion transmembrane transporter activity and release of sequestered calcium ion into cytosol by sarcoplasmic reticulum, (see Additional file 6: Table S6).

In Figure 4A-C the GO biological process, cellular components and molecular functions are depicted.

Note, some of the ontology terms could be grouped, i.e. chaperone-mediated protein complex assembly and folding, endoplasmic reticulum unfolded protein response, ER-nucleus signalling pathway and response to ER oxidative stress as well as hypoxia, blood coagulation, developmental growth and regulation of programmed cell death.

### Cellular components and molecular functions

As depicted in Figure 4B 76 significantly regulated proteins were mapped to 21 cellular components (see Additional file 7: Table S7), i.e. mitochondrial crista, matrix and inner membrane, endoplasmic reticulum lumen, early endosome and cytoplasmic membrane-bounded vesicle, chylomicron, very-low and high density lipoprotein particle, proteasome accessory complex, peroxisome, extracellular vesicular exosome and extracellular membrane-bounded organelle.

Furthermore, 75 significantly regulated proteins were mapped to 21 molecular functions (see Figure 4C) and included arginase activity, fructose-bisphosphate aldolase activity, hydrolase and oxidoreductase activity, acting on carbon-nitrogen (but not peptide) bonds, acting on aldehyde, CH-OH group or oxo group of donors, NAD or NADP as acceptor as well as steroid dehydrogenase activity. In addition, phosphatidylcholine-sterol O-acyltransferase activator activity, cholesterol transporter activity, sterol transporter, antioxidant and lipid transporter activity as well as electron carrier and serine-type endopeptidase inhibitor activity were prominent functions. Finally, proteins functioning in metal ion and purine ribonuleoside triphosphate binding, lipoprotein particle receptor binding, chaperone and oxygen binding, binding of magnesium ion and NAD, protease and single-stranded DNA binding were observed as disease regulated (Additional file 8: Table S8).

### Pathway analysis of tumour proteins

**Figure 5:**
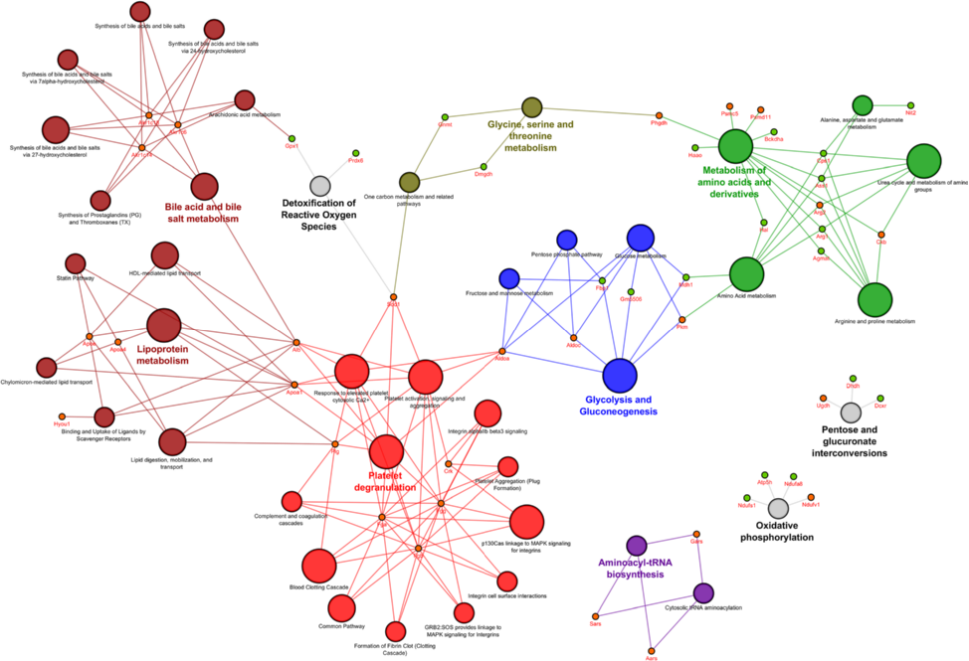
**Molecular interaction and biological pathways networks of regulated proteins in liver tumours of EGF transgenic mice.** Cytoscape 3.0.2 with plugins (see Methods section) are used to generate functionally grouped network of pathways. Grouping of significant pathway terms (p ≤ 0.05) were based on kappa score threshold of 0.4, initial group size of 2 and sharing group percentage of 50. The pathway network consisted of 47 significantly regulated proteins involved in distinct pathways which are colour-coded. Note, the three individual terms are grey-coloured. Up and down-regulated proteins are coded as orange and green small discs, respectively.

In all, 96 significantly regulated proteins were classified by the REACTOME, KEGG and WikiPathway databases, respectively. The different databases provided similar information with the majority of tumour proteins acting in 4 major metabolic pathways (see Figure 5 and information derived from ClueGO and CluePedia). For example, the proteins ALDOA, ALDOC, FBP1 and PKM function in glycolysis and gluconeogenesis whereas AKR1C6, ALDOA, ALDOC and FBP1 are part of the fructose and mannose metabolic pathway. Likewise, ATP5H and NDUFV1 are part of the oxidative phosphorylation pathway and MDH1 and PKM contribute to pyruvate metabolism. Similarly, the proteins AKR1C14, AKR1C18, AKR1C6, ALB, APOA1, APOA4, APOE, FDPS, GPX1, HACL1 and PLG take part in the metabolism of lipids, arachidonic acid and lipoproteins whereas the proteins AGMAT, ARG1, ARG2, BCKDHA, CKB, CPS1, HAAO and PHGDH are specified for arginine and proline metabolism. In the same manner the proteins GPX1, ITPA and NME1 contribute to the metabolism of nucleotides and related to this are the proteins ITPA, PKM and PSMC5 which are part of the purine metabolic pathway. Apart from these pathways a highly significant regulation of the blood coagulation cascade, platelet activation and fibrinolysis was observed as defined by the proteins CRK, FGA, FGB, FGG, PLG and SOD1 all of which were highly significantly regulated. Furthermore, tRNA aminoacylation (AARS, GARS and SARS), advanced glycosylation endproduct receptor signalling (ALB, CAPZA1 and LGALS3), peroxisome (ECH1, HACL1 and SOD1), protein processing in endoplasmic reticulum (GANAB, HYOU1 and PDIA4), proteasome (PSMC5 and PSMD11) and activation of chaperone genes by XBP1(S) and ‘unfolded protein response’ (HYOU1 and LMNA) are pathways significantly perturbed in liver cancer induced by EGF (see Additional file 9: Table S9 and Additional file 10: Table S10).

### Identification of master regulatory proteins

Using the designated workflow of the GeneXplain platform (see Methods section) we searched for master regulatory proteins. The software is designed to identify molecules upstream of regulated tumour proteins to assist in the construction of molecular circuitries. After annotation of the input datasets the tool “Find master regulators in networks (GeneWays)” was used to identify key nodes amongst 54 proteins exclusively expressed in tumours (To). This revealed 24 upstream regulatory molecules. Among them five were selected for their link to the EGFR signalling pathway, i.e. PLAUR, FGFR1, PTBP1 and AGTRAP while the protein S100A1 was chosen for its importance in the PLAUR/EGFR network, (see Additional file 11: Figure S1A-E).

In Additional file 3: Table S3 and Additional file 12: Table S11, the tumour regulated proteins distributed amongst the selected master regulatory molecules are summarized.

**Figure 6:**
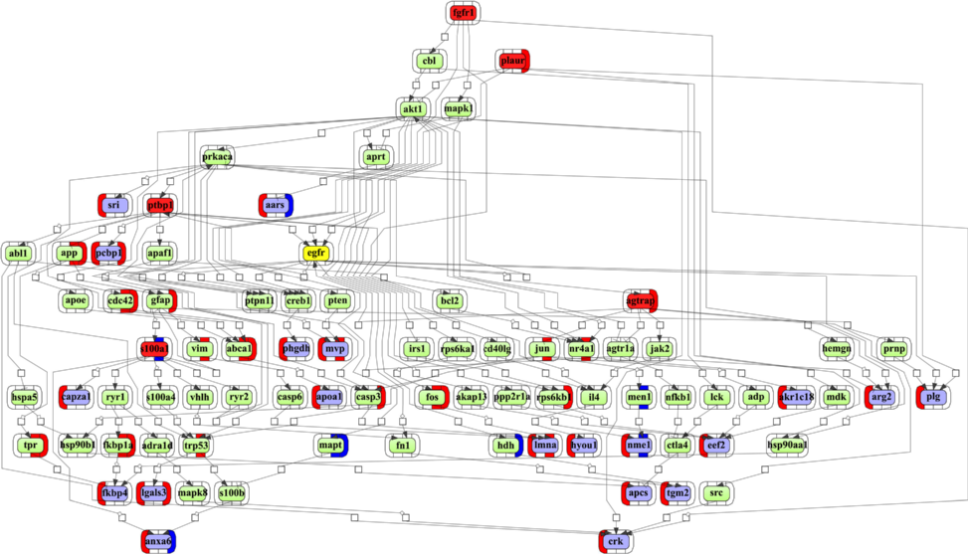
**Integrated master regulatory network for proteins uniquely expressed in tumours.** Based on network information obtained for the 5 different master regulators an integrated hybrid network was constructed. The network contained 82 proteins including 20 with connectivity to EGFR signalling (yellow coloured inner node). The master regulator, the connecting proteins (network elements) and regulated proteins are given as red, green and blue coloured inner node, respectively. Furthermore, each node is partitioned into four segments whereas the first segment seen from left refers to tumour specific proteins and is red-coloured. The second, third and fourth segments refer to either up- and down-regulated proteins, tumour specific gene expression changes and gene regulations in transgenic non-tumour liver tissue, respectively. Increased expression of either proteins or genes is given in red, whereas the blue colour denotes repressed expression.

In support of its biological significance the constructed networks were enriched with gene expression data from transgenic non-tumour and tumour tissues. Thus, the gene and protein data were merged and hybrid networks for each master regulatory protein were constructed. Subsequently, these were merged into one (see Figure 6) and the integrated hybrid network consisted of n = 82 network proteins of which n = 20 were tumour specific. In support, the genes coding for *lmna*, i.e. a component of the nuclear lamina that is frequently up-regulated in cancers and *mvp* that codes for multidrug resistance were up-regulated (ur-T) whereas *nme,* a suppressor of metastasis was repressed in expression (dr-T). Likewise, the genes coding for *igals3*, i.e. a beta-galactoside-binding protein frequently overexpressed in cancers and *pcbp1* that is involved in transcription and functions as an inhibitor of invasion [65] were up-regulated in transgenic non-tumour livers (ur-Tr-nT) whereas transcript expression of *aars*, a member of tRNA synthases and *anaxa6*, a calcium-dependent, phospholipid-binding protein with important roles in the tumour microenvironment and metastasis were repressed (dr-Tr-nT). Finally, the entire network was enriched with expression data of 16 and 17 genes, respectively that were significantly regulated in tumour and non-tumour transgenic livers.

Next, we searched for master regulatory molecules by considering 82 regulated tumour proteins obtained from the comparison tumour specific or up- and down regulated as compared to healthy non-transgenic controls (To + UR + DR). This revealed 29 filtered (threshold radius of 10) upstream regulators. Among these 7 were selected as candidates because of their regulation in liver tumours and their link to EGFR signalling. Notably, in the constructed network all master regulators were significantly up-regulated and included PDIA4, APEH, PEBP1 and APOE while the protein expression of ARG1, FBP1 and HAAO was repressed (see Additional file 13: Figure S2A-G). Note, in the case of ARG1 transcript expression was equally repressed.

In Additional file 3: Table S3 and Additional file 12: Table S11 the tumour regulated proteins distributed amongst the selected master regulatory molecules are summarized.

**Figure 7:**
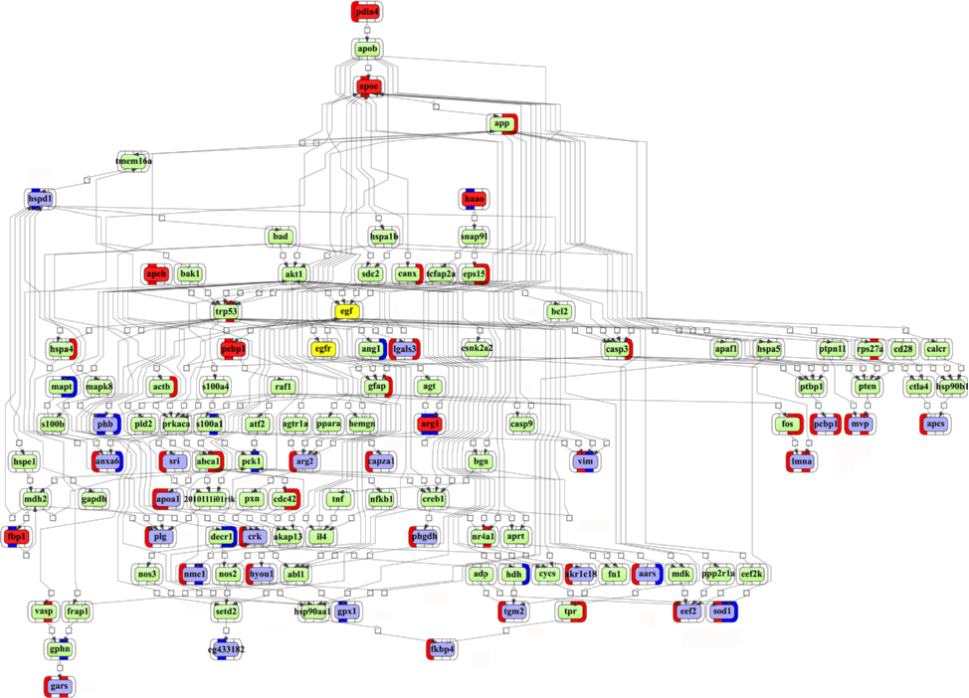
**Integrated master regulatory network for HCC regulated proteins.** Based on network information obtained for 7 different master regulators an integrated hybrid network was constructed. The network contained 114 proteins including 34 with connectivity to EGF/EGFR signalling (yellow coloured inner node). The master regulator, the connecting proteins (network elements) and regulated proteins are given as red, green and blue coloured inner node, respectively. Furthermore, each node is partitioned into four segments whereas the first segment seen from left refers to tumour specific proteins and is red-coloured. The second, third and fourth segments refer to either up- and down-regulated proteins, tumour specific gene expression changes and gene regulations in transgenic non-tumour liver tissue, respectively. Increased expression of either proteins or genes is given in red, whereas the blue colour denotes repressed expression.

In support of its biological significance the fused hybrid network was enriched for gene expression data derived from transgenic non-tumour and tumour tissues. Thus, the integrated hybrid network consisted of 34 out of 82 regulated proteins and gene expression calls evidenced 6 of the 27 up-regulated tumour (To + UR) proteins to be regulated at the transcript level as well whereas among the 7 down-regulated tumour proteins (DR) the gene *arg1* was repressed in expression. Likewise, gene expression data from non-tumour transgenic livers evidenced 5 genes out of 27 networks partners to be increased in expression (ur-Tr-nT) and among the 7 down-regulated networks proteins the gene *phb* was repressed (dr-Tr-nT). Thus, when the tumour gene expression data of the entire network was considered a total of 22 genes were regulated, of which 13 were up-regulated and 9 were repressed in expression, (see Figure 7).

### Protein interaction network

**Figure 8:**
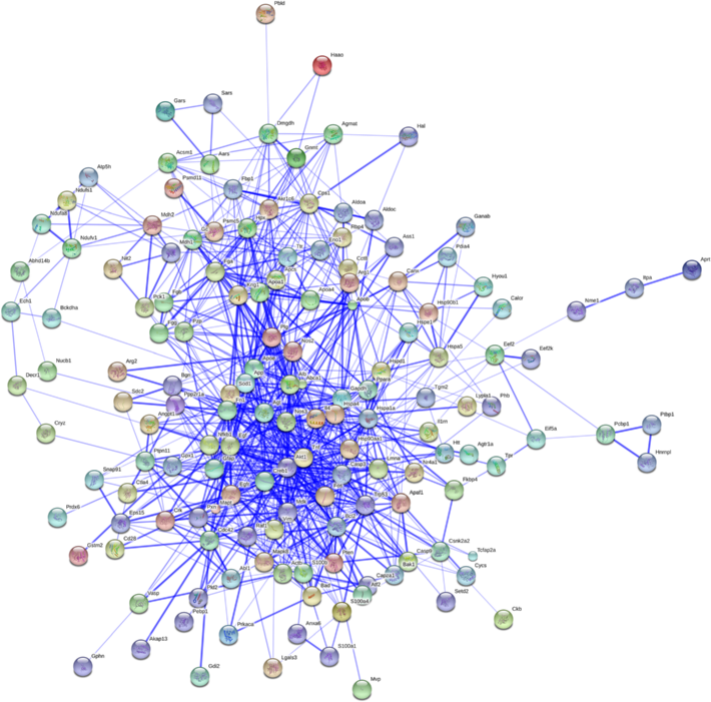
**STRING protein-protein interaction network.** The network consisted of 69 statistically significantly up- and down-regulated proteins and 7 regulated proteins which failed to reach statistical significance. This STRING protein-protein network is a confidence view and a required default confidence score of 0.4 was set. The protein network depicts interaction of regulated proteins including master regulators connected to EGFR signalling.

Based on the information of the hybrid master regulatory network and in addition to other disease regulated proteins summarized in Table 1 (note, some of the proteins were not part of the networks) a total of n = 122 disease regulated proteins were considered for network construction. After filtering for non-connected proteins the STRING database informed on n = 151 protein-interactions of which n = 76 were disease regulated as identified in the present study. Among these 45, 24 and 7 were either up-, down- or not statistically significantly regulated. Furthermore, gene expression calls for 45 up-regulated proteins were supported by 5 up- and 4 down-regulated genes identified in tumours and 4 up- and 6 down-regulated genes in transgenic non-tumour livers. Likewise, gene expression calls for 24 down-regulated proteins were supported by 8 and 5 down-regulated genes in tumours and transgenic non-tumour livers, respectively. Therefore, the entire network was supported by 14 induced and 17 repressed tumour specific gene expression changes and 16 up-regulated and 13 down-regulated genes observed in transgenic non-tumour livers. As depicted in Figure 8 the proteins of the fusion network displayed functional associations via the EGF/EGFR network and included 69 out of 96 (72%) significantly regulated tumour proteins with 6 out of 7 master regulators being connected to EGFR through the network’s proteins (see Additional file 14: Table S12 for possible protein-protein interactions and related scores).

### Pathways mapping of fussed network proteins

**Figure 9:**
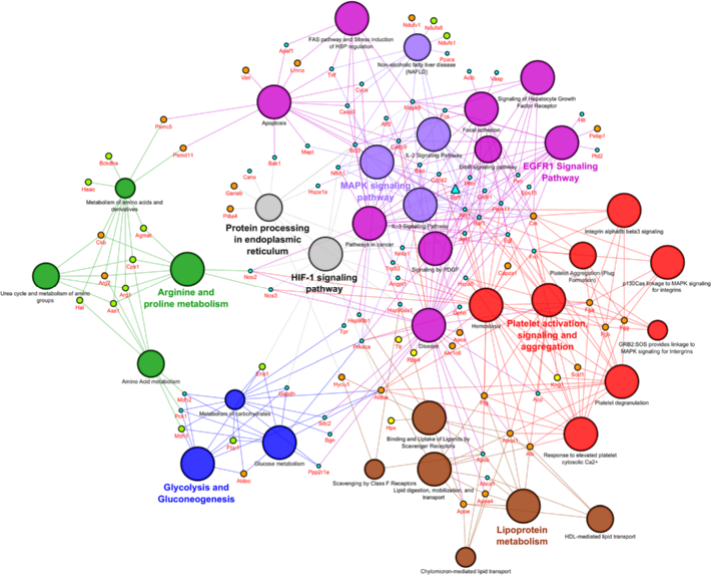
**Pathways mapping of fussed network proteins.** Cytoscape 3.0.2 with plugins (see Methods section) are used to generate functionally grouped network of pathways. Grouping of significant pathway terms (p ≤ 0.05) were based on kappa score threshold of 0.4, initial group size of 2 and sharing group percentage of 50. The pathway network consisted of 35 significantly and 7 non-significantly regulated proteins involved in distinct pathways which are colour-coded. Note, the two individual terms are grey-coloured. Up and down-regulated proteins are coded as orange and green small discs, respectively. Up- and down-regulated as well as non-significantly regulated proteins and connecting proteins of the network are given as orange, green, yellow and blue coloured discs, respectively. The network depicts protein-protein interactions in liver tumours of EGFR transgenic mice and their relation to various pathways under the influence of EGFR signalling. EGFR is highlighted as blue triangle in this network.

Of the 151 network proteins 109 could be mapped to distinct pathways. After removal of non-relevant terms such as Alzheimer disease a total of 94 proteins were mapped to 6 pathways with meaningful associations (see Figure 9) and consisted of ‘platelet activation, signalling and aggregation (platelet degranulation)’, ‘lipoprotein metabolism’, ‘MAPK signalling pathway’, ‘glycolysis and gluconeogenesis’, ‘metabolism of amino acids and derivatives (arginine and proline metabolism)’, ‘apoptosis’ and ‘EGFR1 signalling pathway’. Additionally, a total of 2 and 3 tumour regulated proteins were mapped to the HIF-1 signalling and protein processing in endoplasmic reticulum pathways, respectively. The pathway mapping was also supported by gene expression data with 10 up- and 9 down-regulated genes in tumours and 9 up- and 6 down-regulated genes in transgenic non-tumour livers. Note, two of the significantly regulated tumour proteins, i.e. CRK and PEBP1 are members of the EGFR1 signalling pathway with PEBP1 also functioning as a master regulator while the other regulated proteins are connected to EGFR signalling through cross-talk among the pathways (see Additional file 15: Table S13).

## Discussion

Recent research into the molecular pathogenesis of HCC evidenced significant alterations in signalling pathways. Given the fact that the epidermal growth factor is an important mitogen for hepatocytes we were particularly interested in investigating the consequences of its targeted overexpression in the liver. In our previous study we employed chromatin immunoprecipitation followed by cloning and sequencing of DNA to search for tumour associated gene regulations targeted by novel HNF4alpha P1 and P2 promoter-driven isoforms. This identified EGF-receptor substrate (EPS15R) and EPS15 as regulated by the P2 promoter-driven HNF4alpha splice variant in mouse and human HCC. A molecular circuitry was proposed whereby EPS15 and EPS15R mediate internalization of activated EGFR to stimulate receptor recycling, therefore responding to mitogenic signalling of EGF [66]. In the present study disease proteomics was performed to further investigate the role of EGF in liver cancer. This identified 122 regulated proteins of which 37 are novel and have not been reported so far.

### Extra-cellular space and secreted proteins

A total of 63 proteins were significantly up-regulated (Table 1). Among these 18 were extra-cellular or secreted proteins and included albumin and isoforms of it, apolipoproteins (ApoE, ApoA4 and ApoAI), α-, β-, γ-fibrinogen, plasminogen as well as interleukin 1 receptor antagonist (IL-1RA). Note, an isoform of ApoA1 was already proposed as serum marker of HCC [67] and based on IHC staining IL-1RA expression was confirmed in about 70% of mouse liver adenoma and carcinoma cases; however preneoplastic foci as well as normal hepatocytes surrounding the lesions were negative. Furthermore, RT-PCR analysis confirmed mouse hepatic tumours to contain both secreted and intracellular forms of IL-1ra [51] and serum levels of IL-1ra were monitored to assess therapeutic efficacy of radiofrequency ablation in HCC patients [68].

### Mitochondrial proteins

An important finding of the present study is the statistically significant regulation of 20 mitochondria associated proteins of which 13 were repressed while 7 were up-regulated. Similar results were reported by Chignard and Wei Sun with mitochondrial proteins being the second largest proportion of regulated proteins in human viral HCC [15,69]. Among the repressed proteins were NADH dehydrogenase (ubiquinone) 1 alpha subcomplex 8 and prohibitin, a mitochondrial chaperone. This protein, when deleted (prohibitin KO mice) induced fibrosis, bile duct metaplasia, liver dysplasia and eventually multifocal HCC. However, its overexpression in tumour cell lines inhibited cell proliferation to demonstrate tumour suppressor function [70]. Likewise, glutathione peroxidase 1 (response to oxidative stress) and argininosuccinate synthetase 1 (ASS, urea cycle) were repressed. Note, ASS is the first of two enzymes to convert citrulline to arginine and this pathway allows cells to synthesize arginine from citrulline to function in NO production, ammonia detoxification and synthesis of polyamines. Several reports suggest ASS deficiency to be common in tumour cell lines [25-30], and the present study confirms ASS expression to be confined to healthy non-transgenic control liver, but ASS was absent in tumour tissue extracts (see Table 1). Ablation of ASS in diverse tumours suggests a tumour suppressor function and the fact that forced expression of ASS in osteosarcoma cell lines suppresses growth adds weight to this notion [71].

Another example of tumour specific ablation of proteins refers to glycine N-methyltransferase (GNMT). The enzyme catalyzes the transfer of a methyl group from S-adenosylmethionine (SAM) to glycine thereby generating S-adenosylhomocysteine and N-methylglycine. This protein was completely downregulated in liver tumours. GNMT is known to play a role in the maintenance of genetic stability [44,72], and a novel tumour suppressor function was recently reported that is independent of its catalytic activity but does require its nuclear localization [73].

### Newly identified disease-regulated proteins

Several of the proteins listed in Table 1 were already reported for their tumour specific regulation while proteins so far unknown for their regulations in HCC, are marked with an asterisk (Table 1). These function in diverse biological processes including metabolism, translation and signalling.

Specifically, changes in carbohydrate metabolism are commonly observed in tumours where energy production relies on glycolysis rather than mitochondrial oxidative phosphorylation. In the present study induced expression of several glycolytic enzymes was observed, most notable [1] pyruvate kinase 3 that catalyzes the transfer of a phosphate group from phosphoenolpyruvate to ADP and was shown to be a target of mi-RNA122 in HCC [2,74] aldolase, an enzyme that converts fructose 1,6-bisphosphate into dihydroxyacetone phosphate (DHAP) and glyceraldehyde 3-phosphate and was reported to be a sensitive marker for benign and malignant liver disease [75] and [3] alpha glucosidase 2, a hydrolase that cleaves glycosidic bonds with the release of alpha glucose from carbohydrates.

Further important findings include the tumour specific expression of alanyl-, glycyl- and seryl-tRNA synthetases which catalyze the transfer of specific amino acids to tRNA, as well as regulation of eukaryotic translation elongation factor 2 and Poly(rC) protein 2 that binds to oligo dC. Note, knowledge on the role of aminoacyl-tRNA synthetases in cancer is just emerging [76] and through the use of a lentiviral mediated shRNA vector, a link between aminoacyl-tRNA synthetases [AARS]-interacting multifunctional protein 2 (AIMP2) and repressed EGFR signalling was established that resulted in repressed glucose uptake [77]. We also observed induced expression of heterogeneous ribonucleoprotein (hnRNP) that takes on diverse functions in the processing of mRNA. Its expression was reported to be increased in serum of HCC patients^.^ In contrast, proteins involved in the synthesis and degradation of cholesterol, lipids, steroids and fatty acid were in part oppositely regulated and included induced expression of the aldo-keto reductase family 1. Regulation of this protein has been reported for lung and pancreatic cancers [78], and gene silencing of aldo-keto reductase family 1 B10 resulted in growth inhibition of colorectal cancer cells that might be of therapeutic utility [79]. The repressed expression of certain proteins may also be considered as an adaptive response and includes the enzyme enoyl coenzyme A hydratase 1. Its activity was shown to contribute to lymphatic spread of liver tumours as was evidenced in gene silencing studies [80]. Likewise, we observed repressed expression of dihydrodiol dehydrogenase in tumours. This enzyme plays an important role in the metabolism of steroids that leads to inactivation of circulating androgens, progestins and glucocorticoids and was repeatedly reported to be overexpressed in non-small cell lung cancer. Amongst patients with high DHD expression the incidence of early tumour recurrence and distant metastasis is significantly higher and patients are highly resistant to chemo and radiotherapy [81].

Intriguingly, complete ablation of mitochondrial butyryl coenzyme A synthetase 1, a GTP-dependent lipoate-activating enzyme was observed in tumours of EGF transgenic mice. Little is known about the possible link between butyrate metabolism and liver cancer. However, butyrate is well known to inhibit proliferation of human colon carcinoma cells in an epigenetic manner that involves histone acetylation [82]. Note, it was recently reported that due to the Warburg effect butyrate-mediated histone acetylation and cell proliferation is dictated [83]. Several lines of evidence therefore suggest butyrate to act as a cytosolic sensor for histone acteylation and when transformed to intermediates by butyryl coenzyme A synthetase is unable to escape the mitochondria.

Moreover, we observed a highly significant repression of 2-hydroxyphytanoyl-CoA-lyase. This peroxisomal thiamine pyrophosphate-dependent enzyme is rate limiting in the breakdown of 2-hydroxy fatty acids. The biological role of 2-hydroxy fatty acids has only recently become apparent [84] and cumulative evidence suggests intermediates of energy metabolism to specifically activate G-protein coupled receptors which are now classified as hydroxy carboxylic acid receptors (HCA1-3). The HCA2 receptor is involved in a complex negative feed-back loop whereby ketone bodies derived from fatty acid oxidation are sensed by HCA2 via the activity of 3-hydroxybutyrate that leads to inhibition of lipolysis and to restriction of further fatty acid supply. In this way triglyceride use is diverted and energy demands for tumour growth are met more efficiently. Specifically, during rapid tumour growth and the herewith associated ischemia the yield of high energy bonds (ATP) from glucose oxidation is about twice that of fatty acid oxidation. Our observation that proteins involved in the ß-oxidation of fatty acids were either repressed or unchanged agrees well with this principle (see also discussion below).

The reduced expression of lysophosphopholipase signifies an adaptive response; it catalyses the production of lysophosphatidic acid, i.e. a second messenger known to contribute to tumour cell motility, survival and proliferation [85]. Additionally, the repressed expression of mitochondrial acyl-CoA thioesterase 1 in liver tumours which hydrolyzes acyl-CoAs to free fatty acids and coenzyme A, will influence the supply of ligands for nuclear receptors and the regulation of fatty acid oxidation in mitochondria and peroxisomes. Equally, the regulation of farnesyl diphosphate synthetase, i.e. a key enzyme in the isoprenoid biosynthetic pathway is highly interesting and this enzyme is explored as a drug target of bisphosphonates to treat tumour growth [86]. It’s up-regulation in colon cancers was reported [39]. In the present study repressed expression of the ribosom-compononent RPS12 and enzymes of amino acid metabolism like branched chain ketoacid dehydrogenase E1 as well as dimethyl glycine dehydrogease was observed. Conversely, expression of the proteasome 26S ATPase subunit 5 (p45/SUG) and its non-ATPase regulatory subunit 11 (PSMD11) was confined to tumour tissues (see Table 1); the latter subunit is known to display high activity in embryonic stem cells. This multicomplex molecular machinery degrades intracellular proteins marked up by ubiquitin chains. PSMD11 was reported to be up-regulated in breast cancer cells [87].

Enhanced expression of cytoskeletal proteins such as tubulin β 5 and CAPZA1 was also confirmed by IHC staining (see Figure 3). Differences in the localization of these proteins were obvious with tubulin ß 5 expression being primarily associated with cells proximal to the liver capsule, whereas expression of capping protein Z-line α1 (CAPZA1) was strongly associated with tumour foci and this protein is known to play a pivotal role in cytoskeletal networks to support cell mobility, invasion and metastasis. Additionally, GDI2, a protein functioning in the cycling of Rab GTPases and arginase II, i.e. a non-liver isoform of the urea cycle were up-regulated in tumours of EGF transgenic mice (see Figure 3). Regulation of arginase II was observed in various malignancies including lung cancer [88]. Besides, the actin-binding protein LASP1 was uniquely expressed in tumours and is also up-regulated in breast cancer [89] to possibly support migration of cancer cells [90]. Furthermore, PDIA4, a disulfide bond isomerase and master regulator of the constructed networks (see below) was up-regulated as was kininogen that is part of the blood coagulation system and functions as a precursor of kinin. Conversely, the serinproteinase inhibitor Serpinb1a was repressed in expression to possible limited immunological responses in tumour growth and to influence inflammatory cytokine production by infiltrating monocytes [91].

The significant regulation of the calcium binding protein sorcin and nucleobindin 1 are further highly interesting results. Sorcin is associated with multidrug-resistance in human leukemia cells [92] and nucleobindin 1 is evaluated as a biomarker of colon cancer [93]. In EGF induced liver tumours transthyretin was also up-regulated. This protein is involved in the transport of thyroid hormones and was reported to be aberrantly regulated in thyroid cancer [94]. Among the newly identified proteins is v-crk sarcoma virus CT10. This oncoprotein interacts with several tyrosine-phosphorylated proteins and is part of the intracellular signalling cascades notably the phosphoinositide 3-kinase (PI3K)/AKT pathway [95]. Likewise, regulation of the 170 kDa glucose-regulated protein GRP170 is of great importance. This lumenal endoplasmic reticulum plays a role in immunoglobulin folding as was confirmed by co-immunoprecipitation in four different B cell hybridoma cell lines [11]. In our previous study several immunoglobulins were found to be either repressed or absent in serum of EGF tumour bearing mice and this was particularly obvious for the Ig K and L classes [7]. It remains to be determined whether repression of immunoglobulins can be attributed to aberrant GRP170 activity.

A summary of the biological functions in addition to their previous reported tumour association is given in Additional file 16: Table S14 while the regulation of genes coding for newly identified proteins and of genes coding for commonly regulated proteins in liver tumours and serum of EGF2B-transgenic mice is given in Additional file 17: Table S15 and Additional file 18: Table S16.

### Master regulatory networks

Initially the network construction was based on proteins exclusively expressed in tumours and by selecting master regulatory proteins linked to EGFR signalling. Thereafter, a fused hybrid network was developed in which tumour specific proteins were part of it. Subsequently, the search was extended to all significantly regulated proteins (Table 1). This revealed 7 master regulatory proteins and its associated networks and encompassed 114 proteins of which 34 were disease regulated. Eventually a fused network was developed; however not all disease regulated proteins are part of it. The performed pathway mapping over fused networks (see STRING analysis) defined protein interactions and grouped 76 disease regulated proteins into 6 distinct pathways of which platelet activation, signalling and aggregation is a major one (see Figure 8).

Specifically, the glycoprotein fibrinogen is a multimeric protein and consists of α, ß and y subunits. It is synthesized by hepatocytes and an essential blood coagulation factor with all polypeptide chains being highly regulated in tumours of EGF transgenic mice. Note, an association between coagulation factors and malignancies was established whereby fibrinogen functions as an extracellular matrix protein to interact with integrin receptors in the control of cell proliferation and cell migration [96]. Accordingly, induced gene expression of the integrin receptors *Itgb1*, *Itga3* and *Itgav* was observed in EGF induced liver tumours. In cancer progression a regulatory loop between fibrinogen, platelets and tumour cells has been determined that is activated by platelet cytosolic Ca2+. This second messenger induces integrin receptor complex formation through an association of platelet glycoprotein chains IIb and IIIa (CD41/CD61) thereby creating an active binding site for fibrinogen. An association of tumour regulated proteins with the regulatory loop was confirmed in STRING analysis (Figure 8) and fibrinogen was reported to be an important determinant for metastasis of circulating tumour cells [97]. It is therefore of no surprise that elevated blood fibrinogen is a poor prognostic factor. Haemostatic complications are commonly observed in cancer patients and future therapeutic strategies may focus on the hemostatic system by targeting tumour stroma. In this regard the tumour specific induction of plasminogen is of great importance. This zymogen [98] is converted to plasmin by urokinase (UPA), a serine protease which itself was unchanged; however, gene expression of its receptor was significantly up-regulated in transgenic non-tumour livers. One report suggests the urokinase receptor to prime cells for proliferation in response to EGF by promoting Tyr845 phosphorylation and Stat5b activation; nonetheless, this depended on intracellular c-Src levels [99].

Further studies established a link between induced expression of plasminogen activator, uPA receptor and plasminogen activator inhibitor type-1 (PAI-1) and invasiveness and metastasis of HCC [100,101]. Indeed, a fine balance exists between the plasminogen activating system and its inhibition by PAI-1 and PAI-2. Based on transcriptomic data a highly significant induction of PAI-I (up to 12-fold) in large tumours of EGF transgenic mice was observed [6]; consequently, the regulation of components of the plasminogen activating system may be considered as part of a strategy to degrade extracellular matrix thereby facilitating invasion and metastasis [102,103].

To meet energy demands efficiently different sources are utilized and the induction of the proteins ALDOA, ALDOC, ENO1, PKM and FBP1 is testimony to an altered glycolytic and pentose phosphate pathway. However, with the exception of acyl-CoA thioesterase 2 that was below the limit of detection and functions in the hydrolysis of myristoyl- palmitoyl-, stearoyl- and arachidoyl-CoA esters the regulation of enzymes linked to fatty acid metabolism in mitochondria and peroxisomes was hardly observed.

In pursue of tumour growth and to sustain organelle and membrane biogenesis lipids are de novo synthesized and mobilized from stores and while the complex interaction of hepatic lipid and glucose metabolism in liver disease is the subject of intense research [104] the present study evidences significant regulation of several apolipoproteins, i.e. APOE, APOA1, APOA4 and isoforms of albumin. Apart from lipid transport apolipoproteins play a wider role in cancers and are known to interact with diverse receptors to elicit cellular events as demonstrated for APOE to cause sustained proliferation and survival of cancer cells [105].

A further group of highly regulated proteins are aldo-keto reductases. Their quantitative evaluation in different hepatocellular carcinoma (HCC) cell lines was recently reported [106]. This superfamily of proteins comprises NAD(P)(H)-dependent enzymes which catalyze oxidoreduction of a variety of prostaglandins, steroids and toxic aldehydes. Their involvement in tumorigenesis is supported by several studies and they are explored as drug targets to overcome chemoresistance. In the present study the aldo-keto reductases AKR1C14, AKR1C18 and AKR1C6 were uniquely expressed in tumours, however glutathione peroxidase 1 was repressed to 30% of healthy control livers to possibly support HIF-1 signalling. Indeed, the redox state and therefore glutathione participates in the hypoxic induction of HIF-1 [107], and two proteins of the glycolytic pathway, i.e. ALDOA1 and ENO1, which respond to HIF-1 signalling, were regulated. Moreover, glutathione peroxidase 1 was shifted in the gel as shown in Figure 1 panel G III as a result of post translational modifications that most likely involved c-Abl and Arg kinase activity at Tyr 96 of GPX1 [108]. Likewise, the genes coding for *Aldo1* and *Eno3* were significantly up-regulated in EGF induced liver tumours.

A complex interaction exists between EGFR and RAGE signalling. This receptor for advanced glycation end-products is a member of the immunoglobulin family of cell surface molecules and was reported to significantly influence hepatic tumour growth in murine models of colorectal carcinoma [109]. There is strong evidence for RAGE to promote cancer growth upon ligand dependent activation and several proteins of the S100 family bind to the extracellular domain of RAGE [110,111]. It is of considerable importance that gene expression of *S100a4* and *S100a11* was up to 34-fold induced in tumours of EGF transgenic mice, however expression of *S100a1* was repressed. Likewise the tumour specific expression of the RAGE binding proteins lectin, galactoside-binding, soluble, 3 and CAPZA1 in tumours of EGF transgenic mice is highly suggestive for a sustained crosstalk between RAGE and EGFR [112]. Although the precise mechanism by which S100 proteins stimulate EGFR signalling remains to be elucidated binding of S100A4 to EGF and to other EGFR ligands was reported to possibly facilitate interaction with the receptor [113]. Similarly, the binding of S100A8/A9 to RAGE was shown to promote migration and invasion of human breast cancer cells through actin polymerization and epithelial–mesenchymal transition [114]. Conversely, advanced glycation endproduct (AGE) receptor 1 suppressed oxidant stress-dependent signalling via the EGFR and Shc/Grb2/Ras pathway [115].

As depicted in Figure 5 the amino acid metabolism was another distinct pathway to which several of the regulated proteins could be mapped to. Note, the tumour specific regulations of arginine 1 and 2 as well as the regulation of subunits of the proteasome 26S ATPase (PSMC5 and PSMD11) were already discussed (see above). In the following additional proteins regulated in this pathway are briefly summarized.

Specifically, 3-hydroxyanthranilate-3,4-dioxygenase (Haao) catalyzes oxidation of 3-hydroxyanthranilate to quinolinate and this intermediate functions as a precursor in NAD and pyridine biosynthetic pathways. Expression of Haao was significantly repressed in tumours of EGF transgenic mice and hypermethylation of the coding gene was observed in ovarian cancer [116]. Due to the fact that Haao is significantly repressed at the gene and protein level in at least two different tumour entities (ovarian and liver cancer) the protein may function as a tumour suppressor that appears to be repressed by an epigenetic mechanism.

A significant finding is the tumour specific expression of 3- phosphoglycerate- dehydrogenase which catalyses the production of 3-phosphoglycerate. This intermediate of glycolysis is an essential precursor of the serine biosynthetic pathway. Importantly, a recent metabolomic study evidenced 3-phosphoglycerate to be diverted into serine and glycine metabolism and repressed expression of 3-phosphoglyceratedehydrogenase resulted in impaired tumour cell proliferation [117]. In support of tumour growth the diversion of intermediate of glycolysis affects protein, membrane lipid and nucleotide synthesis.

Moreover, the observed induction of creatine kinase in tumours of EGF transgenic mice creates a circuitry for cellular energy homeostasis in conditions of high metabolic demands [118]. The enzyme catalyses the reversible transfer of phosphate from phosphocreatine to ADP to yield ATP and creatine. Its induction has been observed in many cancers including liver cancer cell lines [119,120] and a further study suggested a possible interplay between p53 mutations, HCC, CK expression with growth-inhibitory effects of cyclocreatine in HCC [121].

While the rationale of tumour cells in embarking on abnormal metabolism had already been discussed (see above) the finding that agmatine ureohydrolase was strongly repressed in EGF induced liver tumours to about 10% of non-transgenic healthy livers is of great importance. This enzyme hydrolyzes agmatine (= decarboxylated arginine) to form putrescine and urea and repression of the enzyme will significantly increase agmatine tissue concentration to influence diverse cellular control mechanisms. Importantly, in the study of Battaglia and coworkers [122] 1 mM agmatine induced large amounts of superoxide production in rat liver mitochondria; however, it did not affect mitochondrial respiration or redox levels of thiols and glutathione. Furthermore, ATP synthesis remained normal and prevented Ca(2+)-induced mitochondrial permeability transition in the presence of phosphate to suggest an intriguing regulatory loop whereby H2O2 induces hypoxia signalling that is linked to abberant metabolism, nonetheless by selecting interconnected physiological pathways tumour cells are equipped to avoid programmed cell death [122,123]. Thus, arginine deprivation is evaluated for its utility in cancer therapy [124].

A further enzyme repressed to 20% of healthy non-transgenic liver is carbamoyl phosphate synthetase 1 (CPS1), i.e. a liver specific ligase to function in ammonia detoxification. It is perplexing that tumour cells disable such an important pathway of the urea cycle. However, a recent study demonstrated DNA hypermethylation as a key mechanism of silencing CPS1 gene expression in human HCC. Note, forced expression of CPS1 induced cell proliferation and the observed repression in human HCC may simply be the result of genomic instability as was observed in tumour cells [125].

## Conclusion

The present study identified novel disease regulated proteins induced by overexpression of EGF to provide new insight into the complex signalling events in HCC. Six major pathways perturbed by EGFR hyperactivity were identified and several of the regulated proteins are interesting drug target candidates and this includes tumour specific expression of kinases as well as proteins involved in aberrant metabolism. An identification of commonly regulated proteins in tumour and sera will be of great utility in the development of biomarkers to monitor disease progression and responses to therapy.

### Additional data files

The following additional data are available with the online version of this paper.

## Additional files

Additional file 1: Table S1. Antibodies and dilutions used to study disease regulation by immunohistochemistry. Additional file 2: Table S2. Statistics and selection criteria for functional grouping of pathways terms of disease regulated proteins. A hypergeometric test followed by bonferroni correction was used with p-value ≤ 0.05. For grouping of pathway terms, the kappa score threshold was set to 0.4. Additional file 3: Table S3. Different master regulators and associated network for proteins expressed in tumours only (To) or significantly regulated when compared to healthy liver of non-transgenic control animals (T + UR + DR). Additional file 4: Table S4. Comparison of disease regulated proteins in mouse and human HCC. Data were taken from ‘The Human Protein Atlas’ database. Additional file 5: Table S5. Comparison of transcriptomic and proteomic data. This comparison revealed 19 significantly regulated genes of which 15 are regulated in common whereas for 4 genes transcript expression was opposite to that of the coded proteins. Additional file 6: Table S6. Biological processes ontology for significantly regulated proteins. Additional file 7: Table S7. Cellular component ontology for significantly regulated proteins. Additional file 8: Table S8. Molecular function ontology for significantly regulated proteins. Additional file 9: Table S9. Biological pathways and their cluster. Cytoscape 3.0.2 with plugins (ClueGO and CluePedia) were used to generate functionally grouped network of pathways based on REACTOME, KEGG and WikiPathways databases. The grouping of significant pathway terms (p ≤ 0.05) is based on a kappa score of 0.4, initial group size of 2 and sharing group percentage of 50. Additional file 10: Table S10. Biological pathways information based each significantly regulated protein (To + UR + DR). This table depicts pathway terms of nearly 80% of regulated proteins (To + UR + DR) and are taken from REACTOME and KEGG databases. Additional file 11: Figure S1. Master regulatory networks for proteins uniquely expressed in tumours with link to EGFR signalling: (A) The PLAUR network consists of 44 proteins of which 20 are tumour-specifically regulated, (B) the FGFR1 consists of 41 proteins of which 18 are tumour-specifically regulated, (C) the PTBP1 network consists of 44 proteins of which 18 are tumour-specifically regulated, (D) the AGTRAP network consists of 43 proteins of which 19 are tumour-specifically regulated and (E) the S100A1 network consists of 38 protein of which 16 are tumour-specifically regulated. Note all networks display connectivity to EGFR signalling (yellow coloured inner node) and in the case of the S100A1 master regulatory protein EGFR signalling is via the PLAUR/EGFR network.The master regulator, the connecting proteins (network elements) and regulated proteins are given as red, green and blue coloured inner node, respectively. Furthermore, each node is partioned into four segments whereas the first segment seen from left refers to tumour specific proteins and is red-coloured. The second, third and fourth segments refer to either up- and down-regulated proteins, tumour specific gene expression changes and gene regulations in transgenic non-tumour liver tissue, respectively. Increased expression of either proteins or genes is given in red, whereas the blue colour denotes repressed expression. Additional file 12: Table S11. Integrated hybrid network with master regulator information for proteins expressed in tumours only (To) and significantly regulated proteins when compared to heathy liver of non-transgenic control animals (T + UR + DR). Additional file 13: Figure S2. Master regulatory networks for regulated proteins with link to EGFR signalling: (A) The PDIA4 network consists of 68 proteins including 32 significantly regulated proteins, (B) the APEH network consists of 68 proteins including 32 significantly regulated proteins, (C) the PEBP1 network consists of 63 proteins including 31 significantly regulated proteins, (D) the APOE network consists of 66 proteins including 31 significantly regulated proteins, (E) the ARG1 network consists of 67 proteins including 31 significantly regulated proteins, (F) the FBP1 network consists of 69 proteins including 31 significantly regulated proteins, (G) the HAAO network consists of 66 proteins including 32 significantly regulated proteins. Note, all networks display connectivity to EGF protein (yellow coloured inner node). The master regulator, the connecting proteins (network elements) and regulated proteins are given as red, green and blue coloured inner node, respectively. Furthermore, each node is partioned into four segments whereas the first segment seen from left refers to tumour specific proteins and is red-coloured. The second, third and fourth segments refer to either up- and down-regulated proteins, tumour specific gene expression changes and gene regulations in transgenic non-tumour liver tissue, respectively. Increased expression of either proteins or genes is given in red, whereas the blue colour denotes repressed expression. Additional file 14: Table S12. Protein interaction information of the fused network. Given are interacting proteins with association score from different prediction methods, i.e. Neighborhood, Gene Fusion, Co-occurrence, Co-expression, Databases and Textmining. Additional file 15: Table S13. Biological pathways and their cluster for fused network proteins. REACTOME, KEGG and WikiPathways database information was used as input data file for Cytoscape 3.0.2. The table shows grouping of significant pathway terms (p ≤ 0.01) and is based on a kappa score of 0.4, initial group size of 2 and sharing group percentage of 50. Additional file 16: Table S14. Biological function of newly identified proteins and their previously reported tumour association. Additional file 17: Table S15. Regulation of genes coding for newly identified proteins in EGF2B-transgenic liver tumours. Additional file 18: Table S16. Regulation of genes coding for common proteins in tumour tissue and serum of EGF transgenic mice.

## Abbreviations

2DE: 2-D Electrophoresis
ACTH: Adrenocorticotropic hormone
AFP: Alpha-fetoprotein
AGE: Advanced glycation endproduct
AGTRAP: Angiotensin II, type I receptor-associated protein
AIMP2: Aminoacyl-tRNA synthetases interacting multifunctional protein 2
Alpha-HCCA: Alpha-Cyano-4-hydroxycinnamic acid
APS: Ammonium persulfate
ASS: Argininosuccinate synthetase 1
ATP: Adenosine triphosphate
CK: Creatine kinase
CK2Α: Casein kinase II subunit alpha
Co: Control specific protein
CPS1: Carbamoyl phosphate synthetase 1
DAB: 3,3′-Diaminobenzidine
DHAP: Dihydroxyacetone phosphate
DR: Down-regulated protein
dr-T: Down-regulated tumour
dr-Tr-nT: Down-regulated transgenic non-tumour
EGF: Epidermal growth factor
EGFR: Epidermal growth factor receptor
EPS15: Epidermal growth factor receptor substrate 15
EPS15R: Epidermal growth factor receptor substrate 15R
ER: Endoplasmic reticulum
ERK: Extracellular signalling regulated kinase
FDR: False discovery rate
FGFR1: Fibroblast growth factor receptor 1
GDP: Guanosine diphosphate
GNMT: Glycine N-methyltransferase
GO: Gene ontology
GRP170: 170 kDa glucose-regulated protein
GTP: Guanosine triphosphate
H_3_PO_4_: Orthophosphoric acid
HCA: Hydroxy carboxylic acid
HCC: Hepatocellular carcinoma
HDAC2: Histone deacetylase 2
HED: Bis(2-hydroxyethyl) disulfide
HRP: Horseradish peroxidase
Ig: Immunoglobulin
IHC: Immunohistochemistry
IL-1RA: Interleukin-1 receptor antagonist
KO: Knockout mouse
LB2: Luria-Bertani medium 2
LB3: Luria-Bertani medium 3
MEK: Mitogen-activated protein kinase kinase
NAD: Nicotinamide adenine dinucleotide
NAD(P)(H): Nicotinamide adenine dinucleotide phosphate
NADP: Nicotinamide adenine dinucleotide phosphate
NO: Nitric oxide
OGP: n-Octyl β-D-glucopyranoside
PAI-1: Plasminogen activator inhibitor type-1
PDGFR: Platelet-derived growth factor receptors
PLAUR: Plasminogen activator, urokinase receptor
PMF: Peptide mass fingerprinting
PTBP1: Polypyrimidine tract binding protein 1
RAGE: Receptor for advanced glycation endproduct
S100A1: S100 calcium binding protein A1
SAM: S-adenosylmethionine
shRNA: Short hairpin RNA
STAT5B: Signal transducer and activator of transcription 5B
TEMED: Tetramethylethylenediamine
To: Tumour specific protein
tRNA: Transfer RNA
Tyr845: Tyrosine residue 845
UPA: Urokinase-type plasminogen activator
UR: Up-regulated protein
ur-T: Up-regulated tumour
ur-Tr-nT: Up-regulated transgenic non-tumour
VEGFR: Vascular endothelial growth factor receptor
XBP1(S): X-box binding protein 1 isoform
