# Supplementary material for: Proteome mapping of epidermal growth factor induced hepatocellular carcinomas identifies novel cell metabolism targets and mitogen activated protein kinase signalling events
Source: BMC Genomics. 2015 Feb 25;16(1):124. doi: 10.1186/s12864-015-1312-z (PMC4357185; doi:10.1186/s12864-015-1312-z)
Supplement: Additional file 16: Table S14. — Biological function of newly identified proteins and their previously reported tumour association. [file 12864_2015_1312_MOESM16_ESM.doc]

**Table S14. Biological function of newly identified proteins and their previously reported tumor association.**

| **No.** | **Protein** | **Protein function** | **Accession number** | **Ratio**  **T/C** | **Tumor association** |
| --- | --- | --- | --- | --- | --- |
| 18* | Aldolase 3 | Carbohydrate metabolism | gi|60687506 | T | occurrance in brain cancer (1) |
| 23* | Alpha glucosidase 2 | Carbohydrate metabolism | gi|26326711 | T |  |
| 9* | Alanyl-tRNA synthetase | Translation | gi|34610207 | T |  |
| 106* | Sars1 protein | Translation | gi|14250361 | T |  |
| 7* | Akr1c12 protein | Fat metabolism | gi|15215042 | 1,9 |  |
| 15* | Aldo-keto reductase family 1, member C14 | Fat metabolism | gi|19527294 | T | Aldo-keto-reductase 1 B10 in human HCC (2) |
| 16* | Aldo-keto reductase family 1, member C6 | Fat metabolism | gi|13487925 | T | Aldo-keto-reductase 1 B10 in human HCC (2) |
| 36* | Butyryl Coenzyme A synthetase 1 | Fat metabolism | gi|16905127 | C |  |
| 49* | Enoyl coenzyme A hydratase 1, peroxisomal | Fat metabolism | gi|7949037 | 0,49 | occurrance in gastric carcinoma (3) |
| 52* | Farnesyl diphosphate synthetase | Fat metabolism | gi|19882207 | T | up-regulated in colon rectal cancer (4) |
| 53* | Fatty acid binding protein 5, epidermal | Fat metabolism | gi|6754450 | 27,9 | occurrance in brain cancer (1) |

| 80* | Lysophospholipase 1 | Fat metabolism | gi|6678760 | 0,25 |  |
| --- | --- | --- | --- | --- | --- |
| 84* | Mitochondrial acyl-CoA thioesterase 1 | Fat metabolism | gi|40538846 | C |  |
| 105* | Rps12 protein | Ribosom component | gi|34849622 | 2 | occurrance in breast cancer (5) |
| 98* | Psmd11 protein | Proteasom component | gi|33585718 | T | occurrance in breast cancer (6) |
| 112* | T43799 proteasome protein p45/SUG [imported] | Proteasom component | gi|11265288 | T |  |
| 4* | 4931406C07Rik [Ester hydrolase C11orf54 homolog] [spot 4342] | Hydrolase | gi|71059921 | 1,5 |  |
| 5* | 4931406C07Rik [Ester hydrolase C11orf54 homolog] [spot 4349] | Hydrolase | gi|71059921 | 1,9 |  |
| 73* | Inosine triphosphatase | Hydrolase | gi|31982664 | T |  |
| 103* | RIKEN cDNA 1810013B01 [abhydrolase domain containing 14b] | Hydrolase | gi|27753960 | 0,6 |  |
| 35* | Branched chain ketoacid dehydrogenase E1, alpha polypeptide | Aminoacid metabolism | gi|31982494 | 0,5 |  |

| 48* | Dmgdh protein [Dimethylglycine dehydrogenase, mitochondrial] | Aminoacid metabolism | gi|12836171 | C |  |
| --- | --- | --- | --- | --- | --- |
| 1* | 170 kDa glucose regulated protein GRP170 Precurser |  | gi|7643979 | T |  |
| 2* | 2 hydroxyphytanoyl-CoA lyase | A peroxisomal enzyme involved in the catabolism of phytanoic acid (7) | gi|18204150 | 0,38 |  |
| 30* | Arginase type II | Arginase converts L-arginine into L-ornithine and urea.(8)  Arginase II is usually not expressed in liver tissue. | gi|6753110 | T | Up-regulated in human pulmonary cancer (9) |
| 46* | Dhdh protein | oxidizes trans-dihydrodiols of aromatic hydrocarbons to the corresponding catechols (10) | gi|21618806 | 0,17 | occurrance in gastric Cancer (11) |
| 47* | Diacetyl/L-xylulose reductase | Aldo-keto reductase | gi|50400594 | C | occurrance in prostate andenocarcinoma (3) |
| 72* | Hypothetical protein  LOC68347 |  | gi|58037115 | 0,39 |  |
| 75* | Interleukin 25 | Cytokine | gi|18250288 | 2,1 |  |
| 76* | Kininogen 1 | Precurser to kinin | gi|12963497 | 2,8 | occurrance in lung adenocarcinoma (12) |
| 82* | Major vault protein | Resistance related protein | gi|12003287 | T | occurrance in lung cancer (13) |
| 90* | Nucb 1 protein | CALNUC [nucleobindin) is an EF-hand, Ca2+-binding protein (14) | gi|49117484 | T | occurrance in colon carcinoma (15) |
| 104* | RIKEN cDNA 2410004H02 |  | gi|26080429 | T |  |
| 109* | Serpinb1a protein | Serinproteaseinhibitor | gi|12834891 | C |  |

| 111* | Sorcin | Ca-binding protein associated with cardiac ryanodine receptors and L-type Ca2+ channels (14) | gi|13385076 | T |  |
| --- | --- | --- | --- | --- | --- |
| 117* | Uap1l1 |  | gi| 28175154 | T |  |
| 120* | v-crk sarcoma virus CT10  oncogene homolog | intracellular signaling cascade and in the activation of the phosphoinositide 3-kinase PI3K/AKT pathway (16) | gi|56205173 | T | Delays apoptoses in P12 renal tumor-zellen (17) |

1. Khalil AA: **Biomarker discovery: a proteomic approach for brain cancer profiling.** *Cancer Sci* 2007, **98:**201-213.
2. Teramoto R, Minagawa H, Honda M, Miyazaki K, Tabuse Y, Kamijo K, Ueda T, Kaneko S: **Protein expression profile characteristic to hepatocellular carcinoma revealed by 2D-DIGE with supervised learning.** *Biochim Biophys Acta* 2008, **1784:**764-772.
3. Kim HK, Park WS, Kang SH, Warda M, Kim N, Ko JH, Prince A, Han J: **Mitochondrial alterations in human gastric carcinoma cell line.** *Am J Physiol Cell Physiol* 2007, **293:**C761-71.
4. Dragani TA, Manenti G, Sacchi MR, Colombo BM, Della Porta G: **Major urinary protein as a negative tumor marker in mouse hepatocarcinogenesis.** *Mol Carcinog* 1989, **2:**355-360.
5. Deng SS, Xing TY, Zhou HY, Xiong RH, Lu YG, Wen B, Liu SQ, Yang HJ: **Comparative proteome analysis of breast cancer and adjacent normal breast tissues in human.** *Genomics Proteomics Bioinformatics* 2006, **4:**165-172.
6. Deng S, Zhou H, Xiong R, Lu Y, Yan D, Xing T, Dong L, Tang E, Yang H: **Over-expression of genes and proteins of ubiquitin specific peptidases (USPs) and proteasome subunits (PSs) in breast cancer tissue observed by the methods of RFDD-PCR and proteomics.** *Breast Cancer Res Treat* 2007, **104:**21-30.
7. Foulon V, Antonenkov VD, Croes K, Waelkens E, Mannaerts GP, Van Veldhoven PP, Casteels M: **Purification, molecular cloning, and expression of 2-hydroxyphytanoyl-CoA lyase, a peroxisomal thiamine pyrophosphate-dependent enzyme that catalyzes the carbon-carbon bond cleavage during alpha-oxidation of 3-methyl-branched fatty acids.** *Proc Natl Acad Sci U S A* 1999, **96:**10039-10044.
8. Wu G, Morris SM,Jr: **Arginine metabolism: nitric oxide and beyond.** *Biochem J* 1998, **336 ( Pt 1):**1-17.
9. Rotondo R, Mastracci L, Piazza T, Barisione G, Fabbi M, Cassanello M, Costa R, Morandi B, Astigiano S, Cesario A, Sormani MP, Ferlazzo G, Grossi F, Ratto GB, Ferrini S, Frumento G: **Arginase 2 is expressed by human lung cancer, but it neither induces immune suppression, nor affects disease progression.** *Int J Cancer* 2008, **123:**1108-1116.
10. Aoki S, Ishikura S, Asada Y, Usami N, Hara A: **Identity of dimeric dihydrodiol dehydrogenase as NADP(+)-dependent D-xylose dehydrogenase in pig liver.** *Chem Biol Interact* 2001, **130-132:**775-784.
11. Chang HC, Chen YL, Chan CP, Yeh KT, Kuo SJ, Ko CJ, Fang HY: **Overexpression of dihydrodiol dehydrogenase as a prognostic marker in resected gastric cancer patients.** *Dig Dis Sci* 2009, **54:**342-347.
12. Al-Dhaheri MH, Shah YM, Basrur V, Pind S, Rowan BG: **Identification of novel proteins induced by estradiol, 4-hydroxytamoxifen and acolbifene in T47D breast cancer cells.** *Steroids* 2006, **71:**966-978.
13. Dingemans AM, van Ark-Otte J, van der Valk P, Apolinario RM, Scheper RJ, Postmus PE, Giaccone G: **Expression of the human major vault protein LRP in human lung cancer samples and normal lung tissues.** *Ann Oncol* 1996, **7:**625-630.
14. Meyers MB, Fischer A, Sun YJ, Lopes CM, Rohacs T, Nakamura TY, Zhou YY, Lee PC, Altschuld RA, McCune SA, Coetzee WA, Fishman GI: **Sorcin regulates excitation-contraction coupling in the heart.** *J Biol Chem* 2003, **278:**28865-28871.
15. Chen Y, Lin P, Qiu S, Peng XX, Looi K, Farquhar MG, Zhang JY: **Autoantibodies to Ca2+ binding protein Calnuc is a potential marker in colon cancer detection.** *Int J Oncol* 2007, **30:**1137-1144.
16. Akagi T, Murata K, Shishido T, Hanafusa H: **v-Crk activates the phosphoinositide 3-kinase/AKT pathway by utilizing focal adhesion kinase and H-Ras.** *Mol Cell Biol* 2002, **22:**7015-7023.
17. Glassman RH, Hempstead BL, Staiano-Coico L, Steiner MG, Hanafusa H, Birge RB: **v-Crk, an effector of the nerve growth factor signaling pathway, delays apoptotic cell death in neurotrophin-deprived PC12 cells.** *Cell Death Differ* 1997, **4:**82-93.
