# Supplementary material for: Proteome mapping of epidermal growth factor induced hepatocellular carcinomas identifies novel cell metabolism targets and mitogen activated protein kinase signalling events
Source: BMC Genomics. 2015 Feb 25;16(1):124. doi: 10.1186/s12864-015-1312-z (PMC4357185; doi:10.1186/s12864-015-1312-z)
Supplement: Additional file 17: Table S15. — Regulation of genes coding for newly identified proteins in EGF2B-transgenic liver tumours. [file 12864_2015_1312_MOESM17_ESM.doc]

**Table S15. Regulation of genes coding for newly identified** proteins in EGF2B-transgenic liver tumors.

| No. | Protein | GI number (NCBI) | Accession number (UniProtKB/Swiss-Prot) | Gene Symbol | Gene Expression Change |
| --- | --- | --- | --- | --- | --- |
| 1* | 170 kDa glucose regulated protein GRP170 precursor | 7643979 | Q9JKR6 | Hyou1 | nf |
| 2* | 2-hydroxyphytanoyl-CoA lyase | 18204150 | Q9QXE0 | Hacl1 | nf |
| 3 | 3-phosphoglycerate dehydrogenase | 52353955 | Q61753 | Phgdh | nf |
| 4* | 4931406C07Rik (Ester hydrolase C11orf54 homolog) (spot 4342) | 71059921 | Q91V76 | 4931406C07Rik | NC |
| 6 | Acylpeptide hydrolase; N-acylaminoacyl peptide hydrolase | 22122789 |  | Apeh | nf |
| 7* | Akr1c12 protein | 15215042 | Q91X42 | Akr1c12 | nf |
| 8 | Akr1c18 protein (aldo-keto reductase family 1, member C18) | 19527284 | Q3U538 / Q8K023 | Akr1c18 | nf |
| 9* | Alanyl-tRNA synthetase | 34610207 | Q8BGQ7 | Aars | NC |
| 12 | Albumin 1 (spot 3707) | 33859506 |  | Alb1 | NC |
| 15* | Aldo-keto reductase family 1, member C14 | 19527294 | Q91WT7 | Akr1c14 | nf |
| 16* | Aldo-keto reductase family 1, member C6 | 13487925 | P70694 | Akr1c6 | NC |
| 17 | Aldolase 1, A isoform | 53733633 |  |  | nf |
| 18* | Aldolase 3 | 60687506 | B1AQE0 / P05063 | Aldoc | nf |
| 21 | Alpha enolase (spot 4501) | 58476212 | Q5FW97 | Eno1 | NC |
| 23* | Alpha glucosidase 2 | 26326711 | Q8BHN3 | Ganab | NC |
| 24 | Annexin A6 | 31981302 | Q3UI56/Q8BSS4 | Anxa6 | nf |
| 25 | Apoa4 protein | 14789706 | Q91XF8 | Apoa4 | NC |
| 26 | Apoe | 71060041 | Q6GTX3 | Apoe | NC |
| 27 | Apolipoprotein A-I (spot 2215) | 26345182 | Q8BPD5 | Apoa1 | NC |
| 28 | Apolipoprotein A-I (spot 3204) | 26345182 |  | Apoa2 | NC |
| 29 | Arginase 1, liver | 7106255 | Q61176 | Arg1 | -2.6 |
| 30* | Arginase type II | 6753110 | O08691 | Arg2 | NC |
| 31 | Argininosuccinate synthetase 1 | 6996911 | Q3UJ34/P16460 | Ass1 | NC |
| 32 | ATP synthase, H+ transporting, mitochondrial F0 complex, subunit d (spot 2120) | 51980458 | Q9DCX2 | Atp5h | nf |
| 34 | Beta 5-tubulin | 18088719 | P07437 | Tubb5 | 2.02 |
| 35* | Branched chain ketoacid dehydrogenase E1, alpha polypeptide | 31982494 |  | Bckdha | -1.73 |
| 36* | Butyryl Coenzyme A synthetase 1 | 16905127 | Q91VA0 | Acsm1 | nf |
| 37 | Cai protein (Pdia4) | 45219865 | Q6NXW4 | Pdia4 | nf |
| 38 | Capping protein alpha 1 subunit | 595917 | P47753 | Capza1 | NC |
| 39 | Carbamoyl-phosphate synthetase 1, mitochondrial | 8393186 | P07756 | Cps1 | nf |
| 40 | Carboxylesterase MH1 | 14331135 | Q8VCT4 | Ces3 | -2.37 |
| 41* | cDNA sequence BC021917 (dihydroxyacetone kinase 2 homolog) | 21703976 | Q8VC30 | Dak | nf |
| 42 | Creatine kinase, brain | 10946574 | Q04447 | Ckb | NC |
| 43 | Cryz protein | 13277837 | P47199 | Cryz | -1.86 |
| 44 | Cu/Zn superoxide dismutase 1513495A | 226471 |  | Sod1 | -1.7 |
| 45 | DEMSMC malate dehydrogenase, cytosolic | 319837 |  | Mdh1 | NC |
| 46* | Dhdh protein | 21618806 | Q8K0E9 | Dhdh | nf |
| 47* | Diacetyl/L-xylulose reductase | 50400594 | Q91X52 | Dcxr | nf |
| 48* | Dmgdh protein (Dimethylglycine dehydrogenase, mitochondrial) | 12836171 | Q9DBT9 | Dmgdh | -2.08 |
| 49* | Enoyl coenzyme A hydratase 1, peroxisomal | 7949037 | O35459 | Ech1 | -2.11 |
| 50 | Eukaryotic translation elongation factor 2 | 33859482 | P58252 | Eef2 | NC |
| 51 | Eukaryotic translation initiation factor 5A (eIF-5A) | 124231 | P10160 | Eif5a | NC |
| 52* | Farnesyl diphosphate synthetase | 19882207 | Q4FJN9/Q920E5 | Fdps | NC |
| 53* | Fatty acid binding protein 5, epidermal | 6754450 | Q05816 | Fabp5 | NC |
| 54 | Fibrinogen, alpha polypeptide | 33563252 | Q99K47 | Fga | nf |
| 55 | Fibrinogen, B beta polypeptide (spot 5602) | 33859809 | Q8K0E8 | Fgb | NC |
| 57 | Fibrinogen, gamma polypeptide | 18044708 | Q8VCM7 | Fgg | NC |
| 58 | FK506 binding protein 4 | 6753882 | P30416 | Fkbp4 | NC |
| 59 | GDP dissociation inhibitor 2 | 38197560 | P50399 | Gdi2 | NC |
| 60 | Glutathione peroxidase 1 | 6680075 |  | Gpx1 | NC |
| 61 | Glutathione S-transferase, mu 2 | 6680121 | P15626 | Gstm2 | NC |
| 62 | Glycine N-methyltransferase (spot 4256) | 34013296 |  | Gnmt | -3.88 |
| 65 | Glycyl-tRNA synthetase | 21264024 | Q9CZD3 | Gars | 3.29 |
| 66 | Haao protein (3-hydroxyanthranilate 3,4-dioxygenase) | 15277547 | Q78JT3 | Haao | nf |
| 67 | Hal protein | 35505393 | P35492 | Hal | -5.66 |
| 68 | Hemopexin | 23956086 | P63039 | Hspd1 | nf |
| 69 | Heterogeneous nuclear ribonucleoprotein L | 33667042 |  | Hnrnpl | nf |
| 70 | HSP60 (spot 2604) | 1334284 |  | Hspd1 | NC |
| 71 | HSP60 (spot 2610) | 1334284 |  | Hspd2 | NC |
| 72* | Hypothetical protein LOC68347 | 58037115 | Q9DCS2 | 0610011F06Rik | NC |
| 73* | Inosine triphosphatase | 31982664 | Q9D892/Q60I30 | Itpa | NC |
| 74 | Interleukin 1 receptor antagonist protein | 238585 | P25085 | Il1rn | 8.58 |
| 75* | Interleukin 25 | 18250288 | A2RSI7 | D17Wsu104e | NC |
| 76* | Kininogen 1 | 12963497 | O08677 | Kng1 | NC |
| 77 | Lamin-A/C | 15929761 | P48678 | Lmna | 2.46 |
| 78 | LIM and SH3 protein 1 | 6754508 | Q61792 | Lasp1 | NC |
| 79 | Liver fructose-1,6-bisphosphatase | 6688689 | Q9QXD6 | Fbp1 | -1.43 |
| 80* | Lysophospholipase 1 | 6678760 | P97823 | Lypla1 | NC |
| 81 | Major urinary protein | 1839508 |  | Mup14 | nf |
| 82* | Major vault protein | 12003287 | Q9EQK5 | Mvp | 1.75 |
| 83 | MAWD binding protein homolog 1 | 31560132 | Q9DCG6 | Pbld | nf |
| 84* | Mitochondrial acyl-CoA thioesterase 1 | 40538846 |  | Acot2 | nf |
| 85 | NADH dehydrogenase (ubiquinone) 1 alpha subcomplex, 8 | 21312012 | A2AL45 | Ndufa8 | NC |
| 86 | NADH dehydrogenase (ubiquinone) Fe-S protein 1 (Ndufs1) | 26331822 | Q91VD9 | Ndufs1 | NC |
| 87 | NADH dehydrogenase (ubiquinone) flavoprotein 1 | 19526814 | Q91YT0 | Ndufv1 | -1.54 |
| 88 | Nit protein 2 (spot 5315) (in large-sized tumors) | 12963555 | Q9JHW2 | Nit2 | nf |
| 90* | Nucb1 protein | 49117484 | Q02819 | Nucb1 | NC |
| 92 | Peroxiredoxin 6 (spot 4207) (in middle-sized tumors) | 6671549 | Q6GT24 | Prdx6 | -1.53 |
| 93 | Phosphatidylethanolamine binding protein | 53236978 | P70296 | Pbp | NC |
| 94 | Plasminogen | 200403 | P20918 | Plg | NC |
| 95 | Poly(rC) binding protein 2; heterogeneous nuclear ribonucleoprotein X | 6754994 | P60335 | Pcbp1 | NC |
| 96 | PREDICTED: agmatine ureohydrolase (agmatinase) | 20848362 |  | Agmat | nf |
| 97 | Prohibitin | 54035592 | P67778 | Phb | NC |
| 98* | Psmd11 protein | 33585718 | Q7TMI0 | Psmd11 | nf |
| 99 | Pyridoxine 5'-phosphate oxidase | 19527238 | Q91XF0 | Pnpo | NC |
| 100 | Pyruvate kinase 3 | 31981562 | P52480 | Pkm2 | NC |
| 101 | Pzp protein (a2-macroglobulin) | 34785996 | Q61838 | Pzp | NC |
| 102 | Retinol binding protein 4, plasma | 33859612 | Q00724 | Rbp4 | NC |
| 103* | RIKEN cDNA 1810013B01 (abhydrolase domain containing 14b) | 27753960 |  | Abhd14b | nf |
| 104* | RIKEN cDNA 2410004H02 | 26080429 | Q571I9 | Aldh16a1 | nf |
| 105* | Rps12 protein | 34849622 | Q6PDW1 | Rps12 | 1.99 |
| 106* | Sars1 protein | 14250361 | P26638 | Sars1 | NC |
| 107 | Selenium binding protein 1 | 22164798 | P17563 | Selenbp1 | NC |
| 108 | Serine (or cysteine) proteinase inhibitor, clade B, member 6a | 6678097 | Q60854/Q4FJQ6 | Serpinb6a | 4.72 |
| 109* | Serpinb1a protein | 12834891 | Q9D154 | Serpinb1a | NC |
| 110 | Serum amyloid P-component | 38174334 | P12246 | Apcs | NC |
| 111* | Sorcin | 13385076 |  | Sri | NC |
| 112* | T43799 proteasome protein p45/SUG [imported] | 11265288 |  | Psmc5 | NC |
| 113 | T-complex protein 1, theta subunit (TCP-1-theta) (CCT-theta) | 12846632 | Q9CS06 | Cct8 | 1.98 |
| 114 | Transglutaminase 2, C polypeptide | 6678329 | P21981 | Tgm2 | NC |
| 115 | Transthyretin | 7305599 | Q5M9K1 | Ttr | NC |
| 116 | Tumor metastatic process-associated protein NM23 | 51980604 |  | Nme1 | -2.11 |
| 117* | Uap1l1 protein | 28175154 | Q3TW96 | Uap1l1 | nf |
| 118 | UDP-glucose dehydrogenase | 6678499 | O70475/Q3TS38 | Ugdh | nf |
| 119 | Unnamed protein product (Galectin-3) | 52987 | P16110 | Lgals3 | NC |
| 120* | v-crk sarcoma virus CT10 oncogene homolog | 56205173 | Q5ND51 | Crk | NC |
| 121 | Vimentin | 31982755 | Q5FWJ3 | Vim | -1.5 |
| 122 | Vitamin D-binding protein | 193446 | P21614 | Gc | NC |

***"nf "****- gene was not present on the MG U74Av2 array.*

***"NC"*** *- no change in gene expression in tumors versus control livers.*

***FC*** *are shown for genes which were significantly up (positive values) or down- (negative values) regulated in tumors.*

*Significance threshold was p-value≤0.05 in T-test for all (n=10) tumors or large (n=3)- or middle (n=4)-sized tumors versus 4 controls.*
