# Supplementary material for: Proteome mapping of epidermal growth factor induced hepatocellular carcinomas identifies novel cell metabolism targets and mitogen activated protein kinase signalling events
Source: BMC Genomics. 2015 Feb 25;16(1):124. doi: 10.1186/s12864-015-1312-z (PMC4357185; doi:10.1186/s12864-015-1312-z)
Supplement: Additional file 18: Table S16. — Regulation of genes coding for common proteins in tumour tissue and serum of EGF transgenic mice. [file 12864_2015_1312_MOESM18_ESM.doc]

**Table S16. Regulation of genes coding for common proteins in tumor tissue and serum of EGF transgenic mice.**

| **Protein** | **Gene Symbol** | **Gene Expression Change** |
| --- | --- | --- |
| Alpha-fetoprotein | Afp | 8,43 |
| Apolipoprotein A1 | Apoa1 | NC |
| Apolipoprotein E | Apoe | NC |
| *Carboxylesterase 3* | *Ces3* | ***-2,37*** |
| Fibrinogen, alpha polypeptide | Fga | nf |
| Fibrinogen, B beta polypeptide | Fgb | NC |
| Fibrinogen, gamma polypeptide | Fgg | NC |
| Major urinary protein | Mup1 | **-2,36** |
| Pzp protein (a2-macroglobulin) | Pzp | NC |
| Serum amyloid P-component | Apcs | NC |

***"nf "****- gene was not present on the MG U74Av2 array.*

***"NC"*** *- no change in gene expression in tumors versus control livers.*

***FC*** *are shown for genes which were significantly up (positive values) or down- (negative values) regulated in tumors.*

Significance threshold was p-value≤0.05 in T-test for all (n=10) tumors or large (n=3)- or middle (n=4)-sized tumors versus 4 control livers.
